# Genome-Wide Analysis of DNA Methylation During Ovule Development of Female-Sterile Rice *fsv1*

**DOI:** 10.1534/g3.117.300243

**Published:** 2017-09-06

**Authors:** Helian Liu, Ya Wu, Aqin Cao, Bigang Mao, Bingran Zhao, Jianbo Wang

**Affiliations:** * State Key Laboratory of Hybrid Rice, College of Life Sciences, Wuhan University, 430072, China; † Hunan Hybrid Rice Research Center, Changsha 410125, China

**Keywords:** rice, female gametophyte abortion, DNA methylation, whole-genome bisulfite sequencing, gene expression and regulation

## Abstract

The regulation of female fertility is an important field of rice sexual reproduction research. DNA methylation is an essential epigenetic modification that dynamically regulates gene expression during development processes. However, few reports have described the methylation profiles of female-sterile rice during ovule development. In this study, ovules were continuously acquired from the beginning of megaspore mother cell meiosis until the mature female gametophyte formation period, and global DNA methylation patterns were compared in the ovules of a high-frequency female-sterile line (*fsv1*) and a wild-type rice line (Gui99) using whole-genome bisulfite sequencing (WGBS). Profiling of the global DNA methylation revealed hypo-methylation, and 3471 significantly differentially methylated regions (DMRs) were observed in *fsv1* ovules compared with Gui99. Based on functional annotation and Kyoto encyclopedia of genes and genomes (KEGG) pathway analysis of differentially methylated genes (DMGs), we observed more DMGs enriched in cellular component, reproduction regulation, metabolic pathway, and other pathways. In particular, many ovule development genes and plant hormone-related genes showed significantly different methylation patterns in the two rice lines, and these differences may provide important clues for revealing the mechanism of female gametophyte abortion.

Rice (*Oryza sativa* L.) not only feeds half of the global population, but it is also useful for basic molecular and genetic studies because of its relatively small genome size and complete genome information (Goff 1999; Delseny *et al.* 2001). As an important participant in the sexual reproduction of rice, the fertility of male and female gametes is directly related to rice yield. In recent years, attention has been focused on the mechanism of male fertility regulation, while few studies have been conducted to elucidate the mechanism of female fertility in rice. The rice ovule is an important sexual reproductive organ, and previous studies have demonstrated the inseparability of ovule development and female gametophyte formation (Reiser and Fischer 1993). Generally, the process of female gametophyte formation is mainly divided into three stages: meiotic division of the megaspore mother cell (MMC), mitotic stage of functional megaspore cells, and mature stage of embryo sac. In detail, the MMC originating from a sporogenous cell derives from below the epidermis of the nucellus and divides into the linear tetrad type after meiosis. Subsequently, the megaspore near the chalaza becomes the functional megaspore, and the remaining three megaspores die during ovule development. The functional megaspore undergoes three rounds of mitotic division and finally forms the female gametophyte with eight nuclei (Yadegari and Drews 2004; Pagnussat *et al.* 2005). The ovule sporophyte tissue provides the necessary nutritional and mechanical support for the formation of the female gametophyte, and communication between the ovule sporophyte and the female gametophyte has been proposed (Bencivenga *et al.* 2011). Formation of the female gametophyte in rice is a complex process that involves multiple genes and thus is influenced by changes in ovule gene expression patterns. Moreover, changes in gene expression in female gametophytes also affect ovule development (Johnston *et al.* 2007; Armenta-Medina *et al.* 2013; Wu *et al.* 2015).

In our experiment, a high-frequency female-sterile mutant rice line (*fsv1*) was derived from a rice cultivar, Gui99, by transferring the genomic DNA of *Panicum maximum* via the ear-stem injecting method. Yang *et al.* (2016) have found that the high-frequency female gametophyte abortion in *fsv1* is caused by the abnormal functional megaspore. Furthermore, many differentially expressed genes and miRNAs involved in ovule development and female gametophyte abortion between *fsv1* and Gui99 have been identified by high-throughput sequencing (Yang *et al.* 2016, 2017). These findings provided important clues for revealing the molecular mechanism of female gametophyte abortion and guided the direction of our research.

DNA methylation plays an important role in regulating plant growth and development, and it is widely involved in the regulation of gene expression, transposon silencing and activation, chromatin-remodeling process, and maintaining the stability of the genome (Kim and Zilberman 2014; Niederhuth and Schmitz 2017). An abnormal DNA methylation status in plants will produce obvious abnormal phenotypes (Ong-Abdullah *et al.* 2015), while some studies have shown that DNA methylation is involved in plant sexual reproduction (Ingouff *et al.* 2017). Generally, plant DNA methylation is mainly distributed in three different sequence contexts: symmetric CG and CHG (hereafter mCG and mCHG) sites and asymmetric CHH (H = A, T, or C) sites (hereafter mCHH). Recently, some techniques have been applied for whole-genome methylation profile analysis, such as methylation-sensitive amplified polymorphisms, methylated DNA immunoprecipitation sequencing (MeDIP-seq), and whole-genome bisulfite sequencing (WGBS), among which WGBS is considered the gold standard for methylation sequencing. The principle of this technique is that nonmethylated cytosine (C) nucleotides are converted to uracil (U) by bisulfite treatment and are read as thymine (T) during sequencing, while methylated cytosines are protected from conversion and are still read as cytosine, thus distinguishing the original methylated from the modified cytosine. Combined with high-throughput sequencing technology and reference sequence alignment, the methylation status of the CG, CHG, and CHH sites can be determined to achieve single-base resolution mapping of the whole-genome DNA methylation map. In addition, methylation in the miRNA gene promoter region is a cause of abnormal miRNA expression regulation. The miRNA is expressed and the target gene is inactivated or inhibited when the promoter region of the miRNA is in the demethylation state. Studies have shown that miRNAs are involved in regulating multiple growth periods of plants, including reproductive development (Xie *et al.* 2015; Zhang *et al.* 2016). Therefore, methylation of the miRNA gene promoter region associated with ovule and female gametophyte development has become the focus of our attention.

In this study, we investigated global DNA methylation alterations in ovules of the rice *fsv1* and Gui99 lines using WGBS. The aim of this work was to explore the DNA methylation patterns and their influence on gene expression during female gametophyte abortion in *fsv1* ovule development. The results will provide important information to characterize the role of DNA methylation in the molecular mechanisms underlying female gametophyte sterility in rice.

## Materials and Methods

### Plant materials

Two lines of rice (*O. sativa* L.), a high-frequency female-sterile rice line (*fsv1*) and a rice wild-type line (Gui99), were used in this experiment. The *fsv1* mutant was obtained by transfusing the genomic DNA of *Panicum maximum* into Gui99 by ear-stem injection. After 36 generations of self-pollination, *fsv1* exhibited stable genetic traits and almost the same genetic background as Gui99. The *fsv1* and Gui99 rice lines had similar morphological characteristics, such as plant height, stem diameter, tiller number, and panicle length. The pollen of *fsv1* was fertile, but ∼80.5% of the female gametophytes were aborted. A detailed description of the materials is provided by Zhao *et al.* (1998) and Yang *et al.* (2016). The rice plants were grown and maintained in the greenhouse of Wuhan University, Wuhan, China.

According to the correspondence between the rice floret morphology and the development of the female gametophyte (Kubo *et al.* 2013), we collected the ovules from meiosis of the MMC stage until the mature embryo sac stage. We removed the ovules from the ovaries with an anatomical needle under a microscope and quickly placed them in liquid nitrogen for DNA extraction. Each sample was subjected to three biological replicates, from each of which was randomly harvested at least 600 florets on 50 plants during the normal growing season.

### DNA library construction and whole-genome bisulfite sequencing

Total genomic DNA was extracted using a Plant Genomic DNA kit (TIANGEN, China) according to the manufacturer’s instructions. Agarose gel electrophoresis and an ultraviolet spectrophotometer were used to evaluate the quality of the genomic DNA. The three biological replicates of genomic DNA were pooled together for each DNA library. The two DNA samples were sonicated to produce DNA fragments using the AIR DNA Fragmentation Kit (Bioo Scientific Corporation). Different fragments of the overhanging structure were treated with T4 DNA polymerase and Klenow enzyme to form blunt ends, followed by the addition of A-tailing to the 3′ blunt ends to produce sticky ends. After end repair and adenylation, the DNA fragments were ligated to an Illumina sequencing primer adaptor. Bisulfite treatment of the genomic DNA was performed using the Zymo EZ DNA Methylation Lightning Kit. With this method, nonmethylated cytosine nucleotides are converted to uracil (U) and read as thymine (T) when sequenced. Methylated cytosines that were protected from conversion were still read as cytosine (C). The bisulfite-treated DNA was purified on a spin column and used to prepare the sequencing library. In this procedure, bisulfite-treated single-stranded DNA was random primed using a polymerase able to read uracil nucleotides to synthesize DNA containing a specific sequence tag. These tags were then used to add Illumina adapters by PCR at the 5′ and 3′ ends of the original DNA strand. The EpiGenome libraries were diluted and loaded onto the cBot DNA Cluster Generation System. After cluster generation was complete, the sample was transferred to the HiSequation 4000 System for sequencing.

### Definition of genome elements

The raw data obtained from Illumina sequencing were first processed to filter out reads containing adapters, unknown or low-quality bases, and then the unique clean reads were mapped to the rice reference genome (https://phytozome.jgi.doe.gov/pz/portal.html#!info?alias=Org_Osativa) using the Alignment software Bowtie 2 (http://bowtie-bio.sourceforge.net/bowtie2/index.shtml). Identification of genome elements was performed according to the method introduced by Li *et al.* (2012).

### Identification of differentially methylated regions (DMRs)

WGBS detected the methylation status of single bases, the number of methylated cytosine loci, and the methylation ratio in each genome element. Significant differences in the DNA methylation ratio in the same genome regions between *fsv1* and Gui99 were evaluated using the χ^2^ test. The false discovery rate (FDR) method was used to determine the threshold of the *P*-value in multiple tests (Benjamini and Yekutieli 2001). DMRs between *fsv1* and Gui99 were considered with an FDR ≤0.001 and at least a 2.0-fold methylation ratio change in genome element sequence counts. The base data manipulations and statistical analysis were performed using the R package.

### Gene ontology (GO), KEGG pathway, MapMan, and clustering analysis

A gene was considered to be a differentially methylated gene (DMG) when at least one DMR was located on this gene. DMGs are the focus of our research, and thus their function was a focus of our attention. Using the Blast2GO software (Version 2.3.5) to obtain GO information for each DMG, we used the WEGO website (http://wego.genomics.org.cn/) to upload the annotation information for each DMG, and the DMGs were classified according to their function. The GO enrichment analysis of the functional significance applies a hyper-geometric test to map all of the DMGs to terms in the GO database, searching for significantly enriched GO terms in the differentially methylated genes compared with the complete genome. The formula used in this analysis has been described by Yang *et al.* (2017).

The KEGG pathway enrichment analysis identifies significantly enriched metabolic pathways or signal transduction pathways in DMGs compared with the whole genome background. The calculation formula and the applied program were the same as that used in the GO analysis. Next, an FDR (Q-value) ≤0.05 was used as the threshold to determine the most significantly enriched pathways in the DMGs.

MapMan software http://mapman.gabipb.org was used to describe the biological pathways associated with the DMGs, as described by Gao *et al.* (2013).

To provide an overview of hyper- or hypo-DMGs, Cluster software and JAVA TreeView software were used to perform the clustering analysis. A document containing the DMGs and their methylation ratios in both samples was uploaded to Gene Cluster 3.0. After data filtering and log transformation, hierarchical clustering of genes and arrays were performed by the average linkage method. Using the cdt. format file generated by the Cluster software with JAVA TreeView software, we can intuitively observe the clustering results of the graphical display.

### Bisulfite sequencing PCR

Genomic DNA was modified using an EZ DNA Methylation Gold kit (Epigenetics) according to the manufacturer’s instructions. In short, 130 μl of the C-T conversion reagent was added to 2 μg of DNA sample and then incubated at 98° for 10 min, 64° for 2.5 hr, and held at 4°. The modified DNA was purified using a Zymo-Spin IC Column (Epigenetics) and stored at −80° until use. Primers for the selected genic sequences were designed using Methyl Primer Express v1.0 and were listed in Supplemental Material, Table S1. For each PCR reaction, 4 μl of bisulfite-treated DNA was used in a 20-μl reaction system. The PCR products were gel-purified using an AxyPrep DNA Gel Extraction Kit (Axygen, Hangzhou, China) and then cloned into the PCRII pGEM-T vector and sequenced. At least 15 clones were sequenced for each sample. The methylation levels were expressed as the percentage (%) per site for each of the three types of cytosines, CG, CHG and CHH, and were calculated by dividing the number of nonconverted cytosines by the total number of cytosines within the assay. Analyses of the bisulfite sequencing results were conducted using the Kismeth website (http://katahdin.mssm.edu/kismeth).

### Data availability

The raw data files obtained in this study by Illumina sequencing have been released in the Sequence Read Archive Database of the National Center for Biotechnology Information. The accession numbers of two sequence read archive runs were SRR5684993 and SRR5684994 (https://trace.ncbi.nlm.nih.gov/Traces/sra/sra.cgi?view=run_browser).

## Results

### Overview of the DNA library sequencing data

To clarify the whole-genome DNA methylation profiles, we sequenced the bisulfite-converted adaptor-ligated genome DNA fragments from developing ovules of *fsv1* and Gui99 rice lines, respectively. The bisulfite conversion efficiency was >99%, providing a reliable guarantee of the accuracy of WGBS. A total of 122.8 million raw reads were generated via Illumina sequencing from the *fsv1* and Gui99 libraries, and then FastQC was applied to assess the quality of the raw data. The results revealed high-quality sequencing (Figure S1). In total, 104.9 million clean reads were obtained after removing the low-quality reads, duplicate reads, and the 5′ and 3′ adapter nulls. Each library contained ∼50 million clean reads, indicating that the depth and density of sequencing were sufficient to provide a high-quality genome-wide methylation analysis. We used Bowtie2 software to map the clean reads to the rice genome, and ∼57% of the reads were uniquely mapped to the rice genome in each sample (Table 1). Only the uniquely aligned reads were used in the following analysis.

**Table 1:** Data generated by WGBS in *fsv1* and Gui99 ovules

Sample	Raw Reads	Clean Reads	Mapped Reads	Mapping Rate (%)	Uniquely Mapped Reads	Unique Mapping Rate	Bisulfite Conversion Rate (%)
Gui99	64633950	55303794	37874308	68.48	31496782	56.95%	99.42
*fsv1*	58193179	49677100	34160912	68.77	28555851	57.48%	99.21

### Genome-wide DNA methylation profiles of fsv1 and Gui99 ovules

By mapping the unique clean reads to the rice genome, we can accurately obtain the methylation status of each cytosine site. Since DNA methylation mainly occurs at three different sequence sites, the CG and CHG sites as well as the CHH sites (H = C, T or A), we calculated the methylation counts and methylation ratio of the three sequence contexts in each genome element by statistical analysis. A total of 1.59 billion cytosine sites were detected throughout the whole genome, of which 21.67% of the cytosine sites were methylated in *fsv1* and 22.56% of the cytosine sites were methylated in Gui99 ovules. For all the sequences, the methylation density of the CG sequence was the highest, followed by the CHG sequence and the CHH sequence (Table 2). The visualization software Circos was used to show the methylation level of the 12 chromosomes in the two rice lines. According to the Circos results, genome elements with high methylation levels were mainly distributed on Ch1, Chr5, and Chr9, while those with low methylation levels were primarily on Chr11 and Chr12 (Figure S2).

**Table 2:** The whole-genome methylation level of *fsv1* and Gui99 ovules

Rice Line	Chr	CpG	mCpG	mCpG%	CHG	mCHG	mCHG%	CHH	mCHH	mCHH%	Total C	Total mC	C%
Gui99	Chr1	3657714	1496039	40.9	3228108	1040813	32.24	11616288	1936072	16.67	18502110	4472924	24.18
	Chr2	2905484	1071684	36.88	2636260	749393	28.43	9679734	1384263	14.30	15221478	3205340	21.06
	Chr3	3021244	1140894	37.76	2724050	777474	28.54	9801969	1492353	15.23	15547263	3410721	21.94
	Chr4	3053058	1174319	38.46	2679200	844076	31.50	9616649	1274372	13.25	15348907	3292767	21.45
	Chr5	2539476	1177823	46.38	2218968	835699	37.66	8104757	1304133	16.09	12863201	3317655	25.79
	Chr6	2594974	1072883	41.34	2295932	764712	33.31	8425977	1269531	15.07	13316883	3107126	23.33
	Chr7	2444714	936023	38.29	2158950	661921	30.66	8021141	1075987	13.41	12624805	2673931	21.18
	Chr8	2314860	1003047	43.33	2061584	722042	35.02	7690127	1132860	14.73	12066571	2857949	23.68
	Chr9	1910938	870619	45.56	1666110	625696	37.55	6205602	984400	15.86	9782650	2480715	25.36
	Chr10	1928208	799691	41.47	1693122	578771	34.18	6263446	863446	13.79	9884776	2241908	22.68
	Chr11	2280308	888872	38.98	2060694	645369	31.32	7836803	946497	12.08	12177805	2480738	20.37
	Chr12	2166398	812002	37.48	1958940	587153	29.97	7452589	881038	11.82	11577927	2280193	19.69
	Total	30817376	12443896	40.57	27381918	8833119	32.53	100715082	14544952	14.36	158914376	35821967	22.56
*fsv1*	Chr1	3657714	1474245	40.3	3228108	1011674	31.34	11616288	1825143	15.71	18502110	4311062	23.30
	Chr2	2905484	1048880	36.10	2636260	724200	27.47	9679734	1303258	13.46	15221478	3076338	20.21
	Chr3	3021244	1118989	37.04	2724050	752182	27.61	9801969	1405329	14.34	15547263	3276500	21.07
	Chr4	3053058	1149040	37.64	2679200	814586	30.40	9616649	1193163	12.41	15348907	3156789	20.57
	Chr5	2539476	1157514	45.58	2218968	812578	36.62	8104757	1221512	15.07	12863201	3191604	24.81
	Chr6	2594974	1051016	40.50	2295932	739905	32.23	8425977	1189074	14.11	13316883	2979995	22.38
	Chr7	2444714	919772	37.62	2158950	642605	29.76	8021141	1010426	12.60	12624805	2572803	20.38
	Chr8	2314860	984233	42.52	2061584	699367	33.92	7690127	1060929	13.80	12066571	2744529	22.74
	Chr9	1910938	856750	44.83	1666110	609409	36.58	6205602	918688	14.80	9782650	2384847	24.38
	Chr10	1928208	783499	40.63	1693122	561018	33.14	6263446	810199	12.94	9884776	2154716	21.80
	Chr11	2280308	870924	38.19	2060694	624862	30.32	7836803	884087	11.28	12177805	2379873	19.54
	Chr12	2166398	795721	36.73	1958940	565231	28.85	7452589	823245	11.05	11577927	2184197	18.87
	Total	30817376	12210583	39.81	27381918	8557617	31.52	100715082	13645053	13.46	158914376	34413253	21.67

### Characterization of DMRs

**Figure 1:**
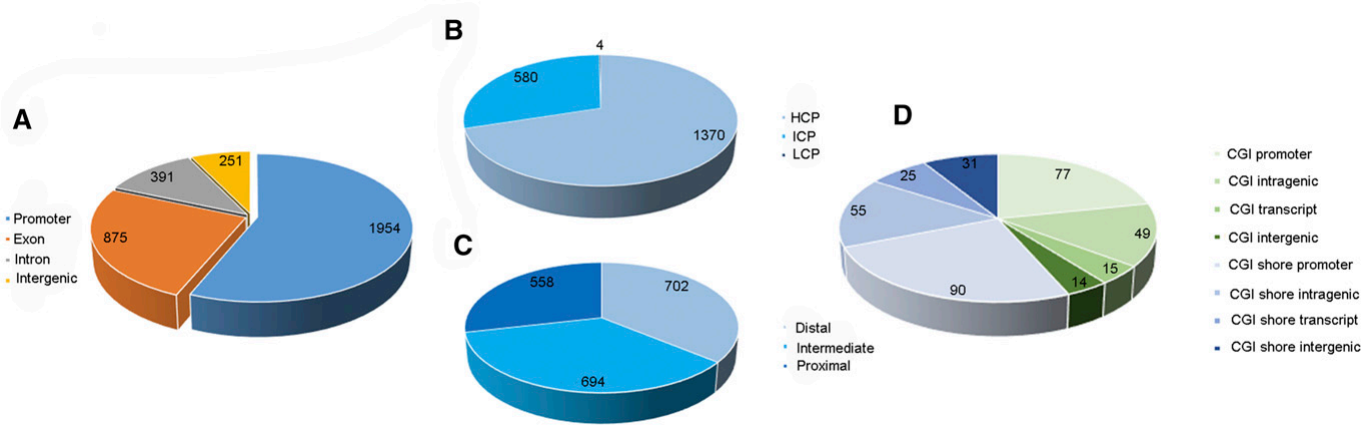
The distribution of DMRs in genome elements. (A) The pie-chart illustrates the overall distribution of genome elements: promoter, exon, intron, and intergenic regions. (B) Pie-chart showing promoter CpG density, divided into high-, intermediate-, or low-density CpG promoters (HCP, ICP, or LCP) as defined in ref. (C) Each promoter of 2700 bp length was divided into three regions: proximal (P; −200 to +500 bp), intermediate (I; −200 to −1000 bp), and distal (D; −1000 to −2200 bp) as described in ref. (D) The proportion of four types of CG island (CGI) and CGI shore.

We used statistics to measure the methylation ratio changes and define DMRs with at least a 2.0-fold change and a FDR ≤0.001. All 3471 DMRs were classified into four types according to genome elements as previously described (Li *et al.* 2012), most of which were distributed in promoter regions (Figure 1A and Table S2). Our data showed that the DMRs occurred more frequently in high CpG promoters (HCPs) than in intermediate CpG promoters (ICPs) and low CpG promoters (LCPs) (Figure 1B). HCPs are thought to be involved in the regulation of housekeeping genes, whereas ICPs are more likely to be *de novo* methylated. However, most LCPs are methylated regardless of whether the genes are silenced or active, suggesting the absence of a correlation between DNA methylation and gene expression in LCPs. Furthermore, the above data indicated that DMRs were more likely to occur in distal (D) and intermediate (I) than in proximal (P) regions in our study (Figure 1C). Gene bodies are mainly composed of exons and introns, and the observed important proportions of DMRs in the first exon and first intron regions indicated that changes in methylation status in regions close to the transcription initiation site may be associated with gene expression. In addition, 251 intergenic regions were detected in the DMRs. Furthermore, many DMRs were located on CG islands (CGIs) and CGI shores, which show a greater distribution in the promoter and intragenic regions, and were more likely to occur on CGI shores compared with CGIs (Figure 1D and Table 3).

**Table 3:** Numbers of DMRs distributed in CGI and CGI shore

Genome Elements	CGI	CGI Shore
Promoter	100	132
Exon	43	47
Intron	12	22
Intergenic	0	0
Total	155	201

### Hyper- or hypo-methylation of DMRs influences neighboring gene expression

**Figure 2:**
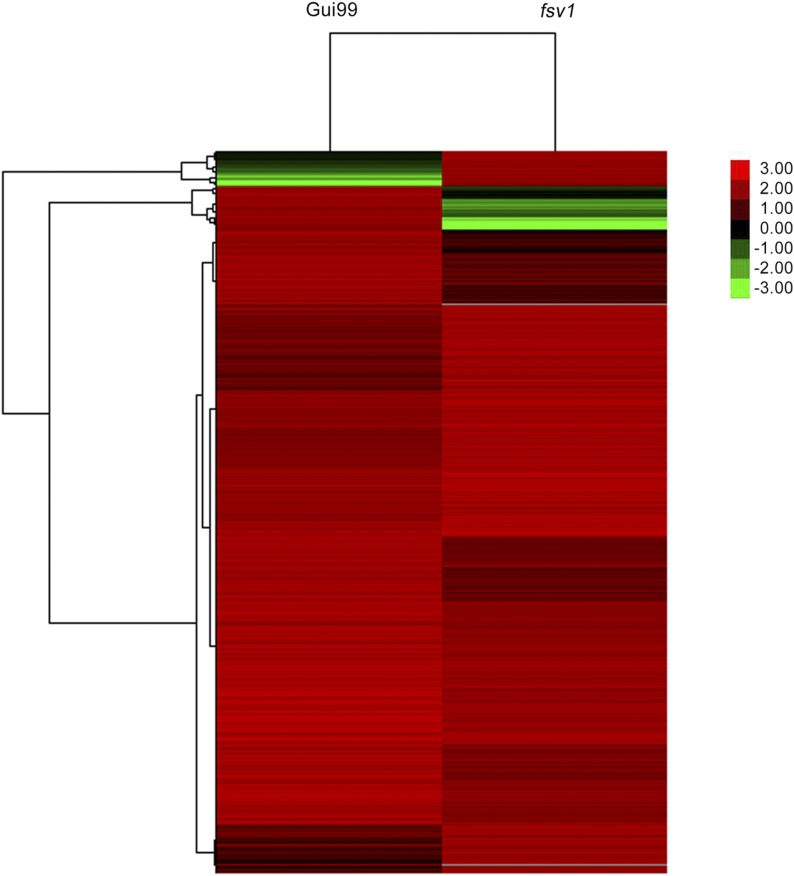
Hierarchical cluster analysis of DMGs in *fsv1* and Gui99. The color key represents methylation ratio normalized log_2_transformed counts. The green color represents lower methylation level and the red color represents higher methylation level. Each column represents an experimental condition, each row represents a gene.

We obtained annotated genome elements for each sample by mapping reads to the rice reference genome, and the promoters, exons, and introns were mapped to the genes. A total of 37,792 genes were found in each sample, of which 36,745 genes were methylated in *fsv1* and 36,802 genes were methylated in Gui99. The standard methylation status of the gene elements in *fsv1* and Gui99 were shown in Figure S3. In this study, genes that overlapped with the sequencing results in the promoter, exon, and intron for the two samples were termed as methylated genes. We screened out 3471 DMRs in gene regions, and they were distributed in 2348 genes, the function of which was the focus of our attention. Genes with similar methylation patterns usually indicate functional correlations. Cluster analysis of gene expression patterns was performed using Cluster and JAVA TreeView software, and gene methylation patterns were clustered using a distance metric based on the Pearson correlation (Figure 2). Hierarchical clustering can provide an overall understanding of DMGs, and the results showed that more DMGs in *fsv1* ovules had a hypo-methylated status.

Compared with the gene methylation status in Gui99 ovules, 1039 genes were hyper-methylated and 1309 genes were hypo-methylated in *fsv1* ovules (Table S2). Most of these DMRs were observed in gene promoters, and many of them were hypo-methylated in *fsv1*. Considering the transcriptome data for *fsv1* and Gui99 ovules (Yang *et al.* 2016), the analysis of the relationship between DNA methylation and gene expression revealed that hyper-methylation or hypo-methylation in some regions was related to the expression of neighboring genes (Table S3). For example, the promoter regions of several genes (*e.g.*, LOC_Os02g10220, LOC_Os04g38950, LOC_Os04g01690, and LOC_Os09g30150, among others) were hyper-methylated in *fsv1*, and the expression levels showed a down-regulation compared with Gui99. As shown in Table S3, 56 DMGs were hyper-methylated in exon regions, of which 33 genes were up-regulated and 23 genes were down-regulated in *fsv1*. The effect of gene body methylation on gene expression was obvious, but its mechanism and correlation remained unclear (Zhang *et al.* 2006; Zilberman *et al.* 2007). Thus, we discovered genes were highly methylated in exon or intron regions, while the expression levels of the genes could be up-regulated or down-regulated.

### GO analysis of DMGs in fsv1 and Gui99 ovules

**Figure 3:**
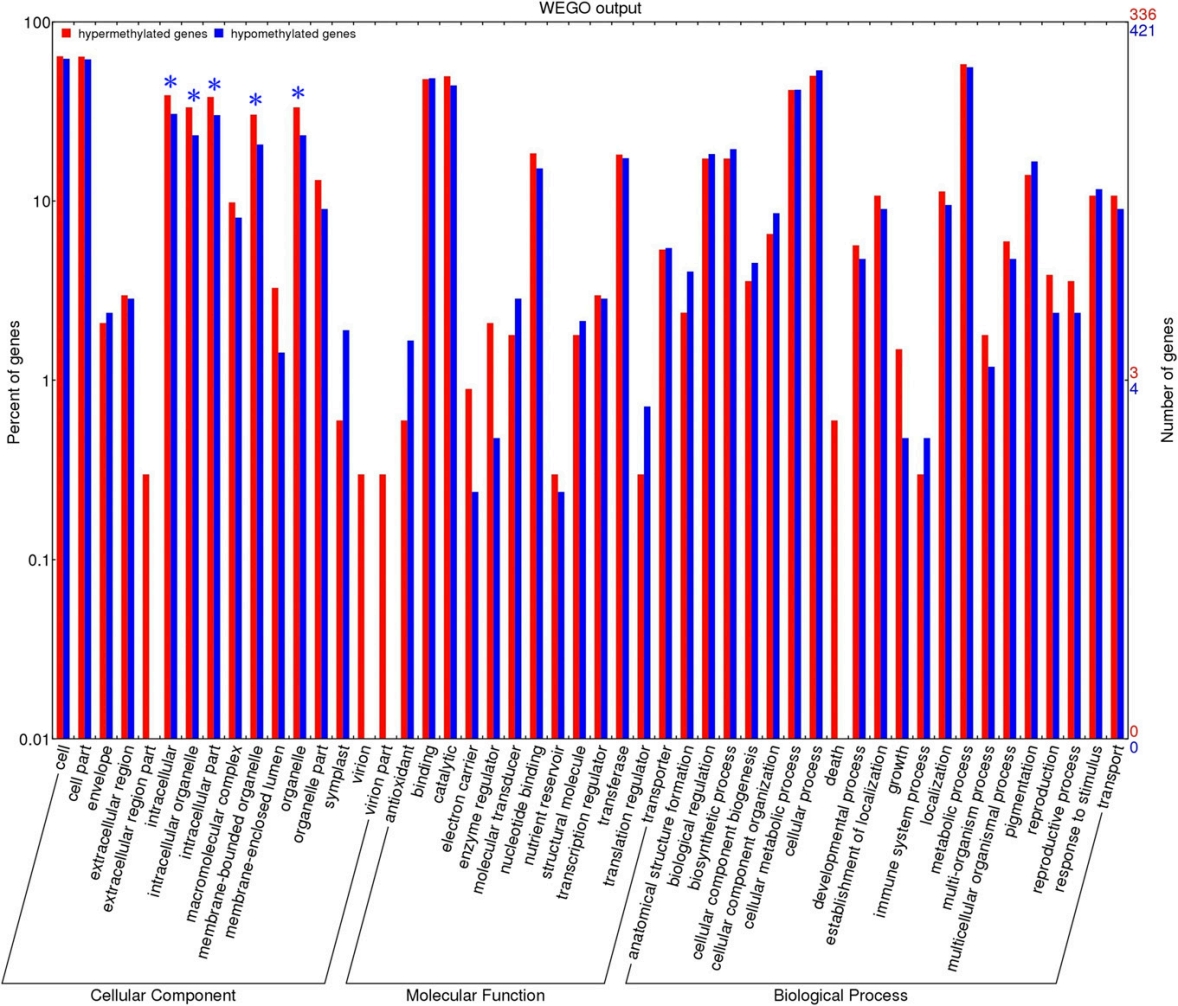
Gene categories and distribution of DMGs between *fsv1* and Gui99. “*” indicates significantly enriched GO terms, of which the *P*-value is below the significant level of 0.05. The figure just displays 50 GO terms and detailed information about the GO analysis is shown in Table S4.

As described above, 2348 genes were found to be DMGs between *fsv1* and Gui99 ovules. We used the WEGO website to functionally categorize the methylated genes and analyze their significant differences. As shown in Table S4, a DMG could be annotated to different GO function terms, and a GO function term could also be annotated for many DMGs. All 757 DMGs out of the 2348 DMGs between *fsv1* and Gui99 ovules were annotated to the GO terms according to the Gene Ontology database (http://www.geneontology.org/), and they were distributed in 236 terms of three main GO categories (Figure 3 and Table S4). Among them, 336 hyper-DMGs and 421 hypo-DMGs were assigned to 217 and 196 GO terms, respectively. The most abundant GO terms in the cellular component, molecular function, and biological process category were cell (GO:0005623), binding (GO:0005488), and metabolic process (GO:0008152), respectively.

The terms organelle (GO:0043226), membrane-bound organelle (GO:0043227), intracellular organelle (GO:0043229), intracellular (GO:0005622), and intracellular part (GO:0044424) were dominant in the cellular component category, and they showed statistically significant differences between the *fsv1* and Gui99 lines, suggesting that organelles and intracellular activities may play important roles in ovule and female gametophyte development. In the molecular function category, it is worth mentioning that the term catalytic (GO: 0003824) was enriched with more cytochrome P450 DMGs, such as *CYP709C9* (LOC_Os07g23570), LOC_Os06g43510, LOC_Os01g52800, LOC_Os02g36150, and LOC_Os03g37080, which will be discussed later. The largest number of functional groups was distributed in the biological process category, and some of the GO terms might be related to female reproductive processes. For example, DMGs assigned to the GO terms reproductive (GO:0022414), cellular process (GO:0009987), cell division (GO:0051301), cell cycle (GO:0007049), chromosome segregation (GO:0007059), megagametogenesis (GO:0009561), and gamete generation (GO:0007276) might participate in the formation of female gametophytes. However, the DMGs in the GO terms cell death (GO:0008219), hormone metabolic (GO:0042445), and gene silencing (GO:0016458) might lead to female gametophyte abortion.

The abortion of female gametophytes is a complex process involving multiple genes, and these GO entries could provide important clues for further studies of mechanisms underlying female gametophyte abortion.

### KEGG and MapMan pathway enrichment analysis of DMGs in fsv1 and Gui99 ovules

**Figure 4:**
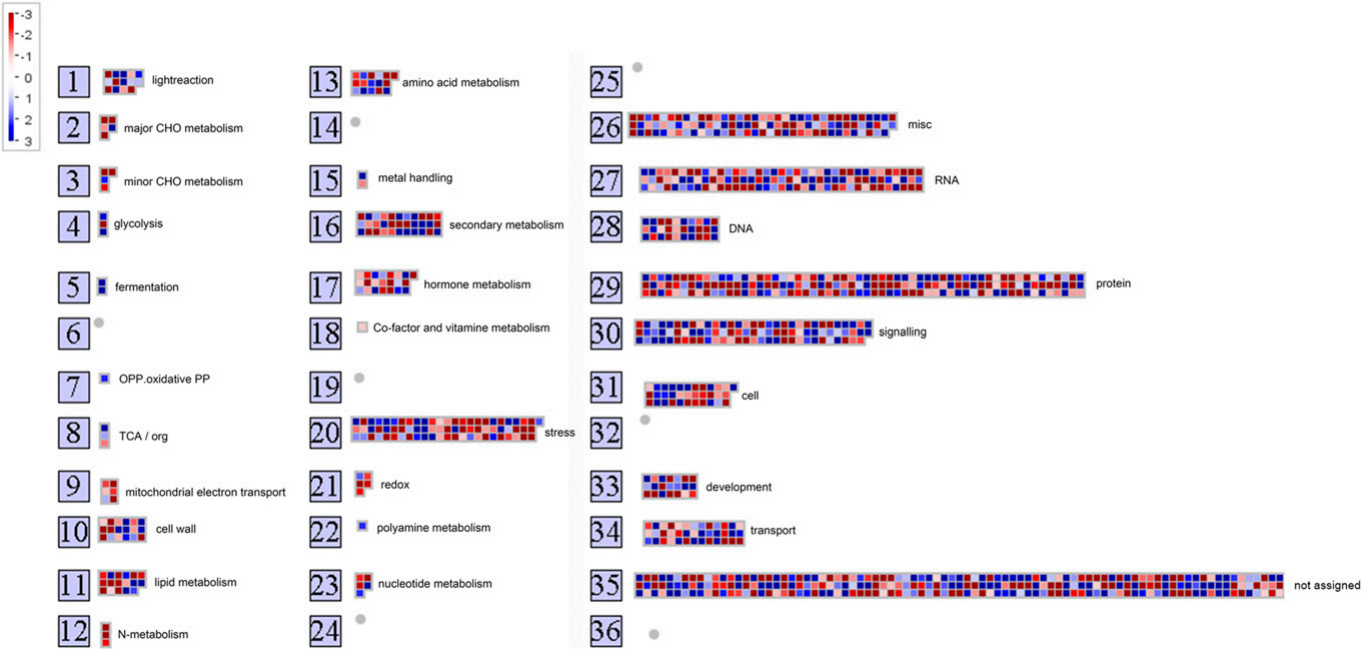
MapMan analysis of 2348 DMGs. Red boxes in these overviews indicate hypo-methylated genes and blue boxes indicate hyper-methylated genes. Detailed information about the overview is shown in Table S5.

To generate further insight about the pathways, we performed KEGG pathway analysis of the DMGs between *fsv1* and Gui99. The major biochemical metabolic pathways and signal transduction pathways of DMGs were identified by KEGG significant enrichment. As a result, 75 pathways were enriched with DMGs, including metabolic pathway, plant hormone signal transduction, biosynthesis of secondary metabolites, and phenylpropanoid biosynthesis (Table 4 and Table S5). In contrast, MapMan software http://mapman.gabipd.org/ was used to describe the DMG-associated biological processes to make these pathways more intuitive and detailed (Figure 4 and Table S6), and the DMGs were enriched in metabolic, protein synthesis and degradation, transcriptional regulation, and hormone metabolism pathways (Figure S4). In plant hormone-related pathways, DMGs that were mainly enriched in auxin, ethylene, and jasmonate-related genes were hypo-methylated, while those related to brassinosteroid were hyper-methylated in *fsv1*.

**Table 4:** The summary of KEGG pathways with the significantly change of methylated cytosine counts in *fsv1* and Gui99

Top 10 of Hyper-methylated Pathways	Top 10 of Hypo-methylated Pathways
Biosynthesis of secondary metabolites (248)	SNARE interactions in vesicular transport (−223)
Pyrimidine metabolism (121)	Metabolic pathways (−203)
Butanoate metabolism (96)	Plant–pathogen interaction (−197)
Ribosome (85)	Protein processing in endoplasmic reticulum (−179)
Ubiquinone and other terpenoid-quinone biosynthesis (82)	Glycerophospholipid metabolism (−145)
Carbon fixation in photosynthetic organisms (80)	Glycerolipid metabolism (−118)
Pantothenate and CoA biosynthesis (77)	RNA transport (−118)
C5-Branched dibasic acid metabolism (77)	Homologous recombination (−117)
Starch and sucrose metabolism (68)	Nitrogen metabolism (−114)
Glycolysis/gluconeogenesis (68)	Phenylalanine metabolism (−112)

Numbers in parentheses represented the total methylated cytosine counts of the DMGs identified in the pathway in *fsv1* minus that in Gui99.

### DNA methylation of transcription factor (TF) gene analysis in fsv1 and Gui99 ovules

TF is a kind of DNA-binding protein that can directly or indirectly recognize or bind to the core sequence of various *cis*-acting elements to regulate the transcriptional efficiency of target genes. TF genes are widely involved in the regulation of plant growth and development. In total, 97 putative TF genes were identified among the 2348 DMGs detected in *fsv1* and Gui99 ovules by blasting against the rice TF database http://planttfdb.cbi.pku.edu.cn/, and they were classified into 28 gene families (Table S7). Compared with Gui99 ovules, the methylation levels of 37 putative TF genes were significantly hyper-methylated in *fsv1*, and the remaining 60 putative TF genes were hypo-methylated. It is worth noting that three putative AP2 family genes, *OsHAP2A* (LOC_Os08g09690), *OsHAP2D* (LOC_Os03g48970), and *OsDREB1G* (LOC_Os08g43210), were hyper-methylated in *fsv1*, while two ARF family genes (LOC_Os01g49120, LOC_Os01g59850) were hypo-methylated. In addition, some TF gene families were assigned to more DMGs, such as the bHLH family, bZIP family, C2H2 family, FAR1 family, MYB family, NAC family, and WRKY family. These TF genes may play important roles in ovule and female gametophyte development, meriting further study.

### Methylation patterns of phytohormone-related genes in the ovules of two rice lines

Phytohormones are well known for their important regulatory roles in plant development. In abortive hazelnut ovules, significant changes occur in indole-3-acetic acid, gibberellins, cytokinin, salicylic acid, abscisic acid, ethylene, and jasmonic acid (Cheng *et al.* 2016). Therefore, the genes involved in the synthesis, transport, and response of these hormones have become the focus of our attention and may play important roles in ovule and female gametophyte development. Based on the KEGG and MapMan analysis results, we found that many phytohormone-related genes were enriched in the plant hormone signal transduction and hormone metabolism pathways. Auxin plays a vital role in plant growth and development and is involved in cell division, elongation, and differentiation (Benková *et al.* 2003). Based on the WGBS data, auxin-related DMGs were detected, among which an auxin efflux carrier family gene LOC_Os01g51780 was hyper-methylated in *fsv1* and another six auxin efflux or auxin responsive family genes (LOC_Os01g48060, LOC_Os03g25289, LOC_Os12g34450, LOC_Os12g33060, LOC_Os03g25289, LOC_Os01g59850) were hypo-methylated. Furthermore, the available evidence indicates that jasmonic acid plays an important role in ovule development (Cheng *et al.* 2016; Hu *et al.* 2016). Based on our data, several jasmonate ZIM domain protein genes were significantly differentially methylated, of which LOC_Os03g27900 and LOC_Os08g33160 were hyper-methylated and LOC_Os07g42370 and LOC_Os07g42370 were hypo-methylated in *fsv1* compared with Gui99. In addition, some important plant hormone-related genes were differentially methylated in *fsv1* ovules. For example, the ethylene sensitivity gene LOC_Os05g46240 and gibberellin oxidase gene LOC_Os05g43880 were hypo-methylated while gibberellin oxidase gene LOC_Os01g55240 was hyper-methylated in *fsv1*.

### MiRNA gene promoter methylation profiles in ovules of fsv1 and Gui99 rice lines

**Figure 5:**
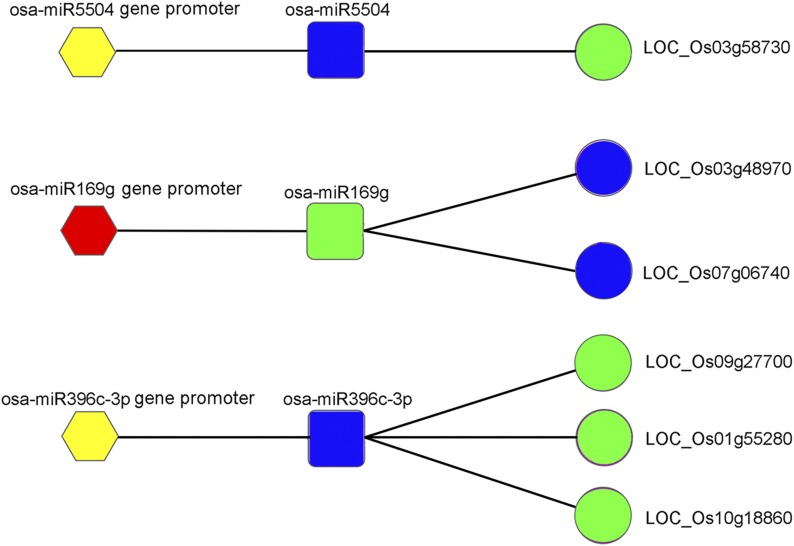
The network of miRNA gene promoter, miRNA, and coherent target genes. Hexagons represent miRNA gene promoter, round squares represent miRNA, and circles represent the coherent target gene of miRNA. Red and yellow represent hyper-methylated and hypo-methylated, respectively. Blue and green represent up- and down-regulated expression level, respectively.

MiRNAs are a class of noncoding small RNAs with a length of 20–24 nucleotides that are important negative regulatory factors and extensively involved in the regulation of plant growth and development (He and Hannon 2004). Eukaryotic gene promoters are important regulatory elements that are indispensable for gene expression, including transcription initiation sites, TATA boxes, and upstream *cis*-acting elements. The upstream promoter of the miRNA gene is closely related to the tissue and the developmental period expression specificity of the plant miRNA. The abnormal methylation status of the miRNA gene promoter may affect the synthesis of miRNAs and affect the expression of its target genes, leading to abnormal growth and development of the plant. Based on our results, 84 differentially methylated miRNA gene promoters were detected in *fsv1* and Gui99 ovules (Table 5), of which 37 were hyper-methylated (for example, miR156i, miR166b-3p, miR166b-5p, miR166e-3p, miR166e-5p, miR169o, miR169g, miR169r-3p, miR169r-5p, miR172a, miR1432-3p, miR1432-5p, miR5535, among others), and 47 were hypo-methylated (miR160c-5p, miR166c-3p, miR166d-3p, miR166d-5p, miR167b, miR167f, miR171d-5p, miR171d-3p, miR1425-3p, miR1425-5p, miR5339, miR5491, miR5810, among others) in *fsv1* ovules. Combined with miRNA and RNA-Seq analyses (Yang *et al.* 2016, 2017), we found that abnormal methylation of these miRNA gene promoters could lead to transcriptional abnormalities in miRNAs, and the abnormal expression of miRNA affected the expression of their coherent target genes (Figure 5). For example, the miR5504 gene promoter was hypo-methylated in *fsv1*, then we found that the expression level of miR5504 was up-regulated in miRNA analyze and the expression level of its coherent target gene LOC_Os03g48970 was down-regulated in *fsv1* of RNA-Seq.

**Table 5:** MiRNA promoters with significantly differentially methylated status between ovules of two rice lines

MiRNA Name	Log2 Fold-Change	Methylation Level	*P*-value	Significance
osa-miR1425-3p	−3.044394119	Hypo-methylation	3.3523E−06	**
osa-miR1425-5p	−3.087462841	Hypo-methylation	2.0804E−06	**
osa-miR1432-3p	1.874469118	Hyper-methylation	0.026893	*
osa-miR1432-3p	1.044394119	Hyper-methylation	0.00027986	**
osa-miR1432-5p	1.736965594	Hyper-methylation	0.044044	*
osa-miR1435	1.169925001	Hyper-methylation	0.039095	*
osa-miR156i	3	Hyper-methylation	0.016686	*
osa-miR159d	−2.807354922	Hypo-methylation	0.0034877	**
osa-miR160c-3p	−1.144389909	Hypo-methylation	0.0052627	**
osa-miR160c-5p	−1.10433666	Hypo-methylation	0.0061281	**
osa-miR166b-3p	1.502500341	Hyper-methylation	0.016861	*
osa-miR166b-5p	1.736965594	Hyper-methylation	0.0044014	**
osa-miR166c-3p	−3.169925001	Hypo-methylation	0.013607	*
osa-miR166d-3p	−3	Hypo-methylation	0.022957	*
osa-miR166d-5p	−3	Hypo-methylation	0.022957	*
osa-miR166e-3p	1.584962501	Hyper-methylation	0.0059267	**
osa-miR166e-5p	1.584962501	Hyper-methylation	0.0059267	**
osa-miR167b	−1.125530882	Hypo-methylation	0.037497	*
osa-miR167f	−1.263034406	Hypo-methylation	0.022275	*
osa-miR169g	2	Hyper-methylation	0.0055546	**
osa-miR169o	2.321928095	Hyper-methylation	0.017365	*
osa-miR169r-3p	1.807354922	Hyper-methylation	0.014604	*
osa-miR169r-5p	1.378511623	Hyper-methylation	0.048778	*
osa-miR171c-3p	2.700439718	Hyper-methylation	0.0035233	**
osa-miR171c-5p	2.584962501	Hyper-methylation	0.0060011	**
osa-miR171d-3p	−2.321928095	Hypo-methylation	0.025026	*
osa-miR171d-5p	−3.321928095	Hypo-methylation	0.0080917	**
osa-miR172a	1.415037499	Hyper-methylation	0.0016432	**
osa-miR1846c-3p	−1.044394119	Hypo-methylation	0.022017	*
osa-miR1846c-5p	−1.044394119	Hypo-methylation	0.022017	*
osa-miR1848	−1.754887502	Hypo-methylation	0.0019694	**
osa-miR1850.2	1.321928095	Hyper-methylation	0.039814	*
osa-miR1850.3	1.321928095	Hyper-methylation	0.039814	*
osa-miR1859	1.584962501	Hyper-methylation	0.037516	*
osa-miR2096-3p	−2.402098444	Hypo-methylation	0.000011225	**
osa-miR2100-3p	1.192645078	Hyper-methylation	0.048544	*
osa-miR2100-5p	−3.169925001	Hypo-methylation	0.013607	*
osa-miR2275a	2.129283017	Hyper-methylation	0.000021442	**
osa-miR2863b	1.736965594	Hyper-methylation	0.044044	*
osa-miR2873a	2.906890596	Hyper-methylation	0.001213	**
osa-miR2907c	−1	Hypo-methylation	0.0018074	**
osa-miR2919	1.286881148	Hyper-methylation	0.00004735	**
osa-miR2932	−1.584962501	Hypo-methylation	0.011048	*
osa-miR319a-3p.2-3p	−1.169925001	Hypo-methylation	0.022704	*
osa-miR319a-3p.2-3p	−1.169925001	Hypo-methylation	0.022704	*
osa-miR396b-3p	−1.584962501	Hypo-methylation	0.0065944	**
osa-miR396b-5p	−1.222392421	Hypo-methylation	0.016184	*
osa-miR396c-3p	−2.584962501	Hypo-methylation	0.0093555	**
osa-miR396d	−1.201633861	Hypo-methylation	0.031607	*
osa-miR3981-3p	1.137503524	Hyper-methylation	0.025448	*
osa-miR3981-5p	1.070389328	Hyper-methylation	0.036884	*
osa-miR399c	−1.485426827	Hypo-methylation	0.047871	*
osa-miR399g	−1.247927513	Hypo-methylation	0.043997	*
osa-miR399j	−2.169925001	Hypo-methylation	0.040849	*
osa-miR415	−1.700439718	Hypo-methylation	0.035614	*
osa-miR439c	−1.502500341	Hypo-methylation	0.0018721	**
osa-miR439d	−1.874469118	Hypo-methylation	0.00003647	**
osa-miR5071	−1.321928095	Hypo-methylation	0.030533	*
osa-miR5071	−2.321928095	Hypo-methylation	0.025026	*
osa-miR5145	1.395928676	Hyper-methylation	0.000096668	**
osa-miR5162	−1.440572591	Hypo-methylation	0.024287	*
osa-miR5339	−4.058893689	Hypo-methylation	2.731E−10	**
osa-miR5489	2.201633861	Hyper-methylation	0.00045157	**
osa-miR5491	−2.584962501	Hypo-methylation	0.001458	**
osa-miR5504	−1.874469118	Hypo-methylation	0.039005	*
osa-miR5521	2.222392421	Hyper-methylation	1.8915E−06	**
osa-miR5527	−1.029747343	Hypo-methylation	0.0058305	**
osa-miR5533	−1.807354922	Hypo-methylation	0.0013048	**
osa-miR5534b	1.184424571	Hyper-methylation	0.014149	*
osa-miR5535	4	Hyper-methylation	0.00019844	**
osa-miR5788	−1	Hypo-methylation	0.0066162	**
osa-miR5789	1.906890596	Hyper-methylation	0.0090287	**
osa-miR5791	−1.157541277	Hypo-methylation	0.01925	*
osa-miR5810	−2.187627003	Hypo-methylation	0.000011529	**
osa-miR5811	−1.874469118	Hypo-methylation	0.039005	*
osa-miR5816	−1.222392421	Hypo-methylation	0.007174	**
osa-miR5822	−1	Hypo-methylation	0.0091971	**
osa-miR6251	3.321928095	Hyper-methylation	0.005432	**
osa-miR812b	1.807354922	Hyper-methylation	0.0027809	**
osa-miR812c	1.362570079	Hyper-methylation	0.021442	*
osa-miR812e	−2.099535674	Hypo-methylation	0.00025097	**
osa-miR812j	3.392317423	Hyper-methylation	0.000049436	**
osa-miR812s	−1.362570079	Hypo-methylation	0.035634	*
osa-miR821a	1.169925001	Hyper-methylation	0.039095	*

In this study, the significantly differentially methylated miRNA gene promoters between *fsv1* and Gui99 were labeled with “**” and “*.” **, |fold change log2| > 1 and *P*-value <0.01; *, |fold change log2| > 1 and 0.01 < *P*-value <0.05.

### Validation of the WGBS data by bisulfite sequencing

**Figure 6:**
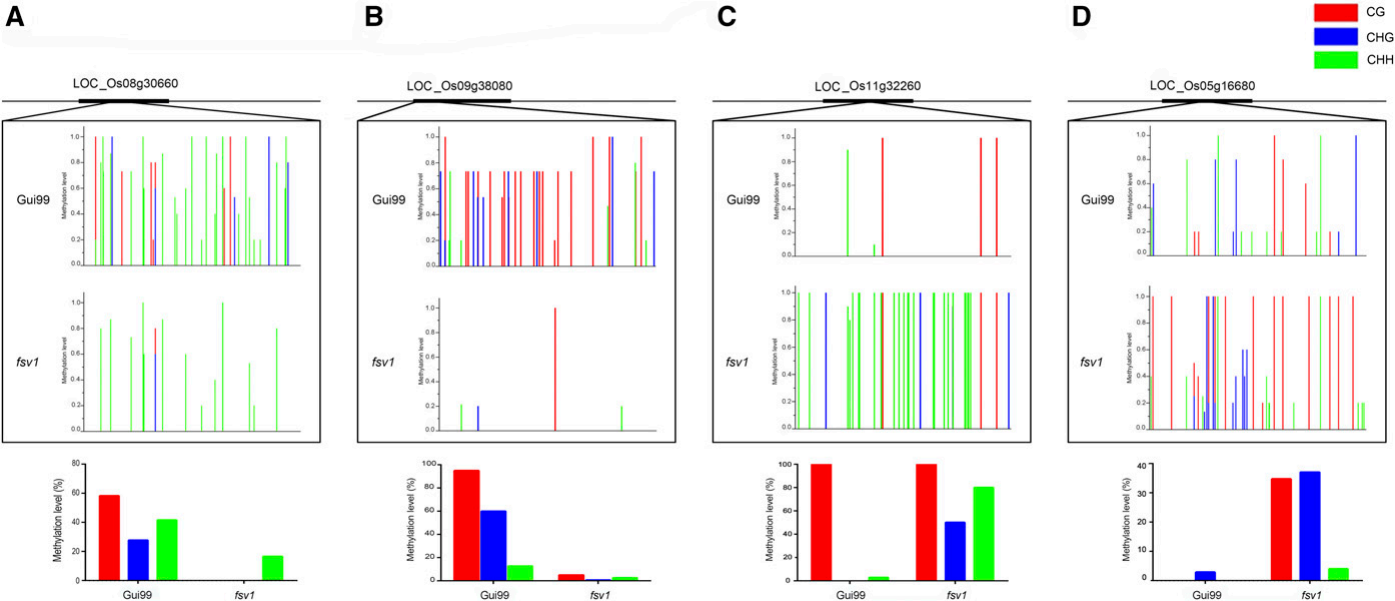
DNA methylation patterns of four DMR-associated genes. (A) Bisulfite sequencing analysis of LOC_Os08g30660 (NB-ARC domain-containing disease resistance protein). (B) Bisulfite sequencing analysis of LOC_Os09g38080 (retrotransposon protein). (C) Bisulfite sequencing analysis of LOC_Os11g32260 (lysosomal α-mannosidase precursor). (D) Bisulfite sequencing analysis of LOC_Os05g16680 (retrotransposon protein). The horizontal axis of the histograms in the top panel represents the location of the various types of cytosine in the sequence; the red, blue, and green columns refer to the collective methylation levels of CG, CHG, and CHH in 15 clones. The histograms in the bottom panel represent the methylation ratio of CG, CHG, and CHH detected by WGBS in the sequence.

In this study, the DNA methylation patterns of 10 DMR-associated genes were randomly selected to carry out bisulfite sequencing to validate the WGBS data (Figure S5), including LOC_Os08g30660 (NB-ARC domain-containing disease resistance protein), LOC_Os12g41890 (amino acid transporter 1), LOC_Os06g47350 (RNA polymerase I specific transcription initiation factor RRN3 protein), LOC_Os10g34490 (Nucleotide-sugar transporter family protein), and LOC_Os12g08300 (glycosyltransferase family protein 2), among others (Figure 6, A–D). We found that more genes were hypo-methylated in *fsv1* compared with Gui99, and the results were almost consistent with the WGBS data. For example, LOC_Os01g17130 (LRR and NB-ARC domains-containing disease resistance protein), LOC_Os12g31180 (retrotransposon protein), and LOC_Os10g34490 (nucleotide-sugar transporter family protein) showed lower methylation levels in *fsv1*. However, LOC_Os05g16680 (retrotransposon protein) and LOC_Os12g41890 (amino acid transporter 1) were hyper-methylated in *fsv1* ovules.

## Discussion

DNA methylation plays an important regulatory role in plant development and fertility, and whole-genome DNA methylation surveys have been performed in thermo-sensitive male sterile rice and other plants (Vining *et al.* 2012; Hu *et al.* 2015; Ausin *et al.* 2016), while few studies have been reported about the methylation profiles of female-sterile rice lines (for example *fsv1*) during female gametophyte development. The objective of the present study was to perform a genome-wide identification of methylated genes that affect ovule development and female gametophyte formation in *fsv1*. To confirm the results obtained from WGBS, the methylation patterns of 10 gene regions in each sample were analyzed by bisulfite sequencing, and the methylation levels of the samples obtained by the two methods were basically consistent, indicating that the high-throughput bisulfite sequencing results were reliable.

DNA methylation plays an important role in gene expression and regulates plant growth and development. The ovules of the female-sterile rice line *fsv1* displayed a slightly lower whole-genome methylation level than Gui99, while localized regions of the genome showed a significantly varied hyper-methylation or hypo-methylation status in *fsv1*. The abnormal methylation status in gene elements may lead to changes in gene expression levels. Generally, promoter methylation is often linked to the suppression of gene expression, whereas the role of gene body methylation is far more uncertain, demonstrating both positive and negative relationships to gene expression (Zhang *et al.* 2006; Zilberman *et al.* 2007). The development of the female gametophyte involves precise and complex gene activities, and changes in the expression levels of individual genes may lead to female gametophyte abortion (Kubo *et al.* 2013). Previous studies have shown that genes that regulate the female gametophyte may be derived from genes expressed by the female gametophyte itself, and may also be derived from genes expressed in the ovule sporophyte tissue (Tucker *et al.* 2012; Song and Chen 2015).

In the rice mutant *OsAPC6*, female gametophyte development is abnormal. The abnormal mitotic divisions during megagametogenesis can be attributed to the inactivation of APC6/CDC16 of the anaphase-promoting complex in rice, which is responsible for cell cycle progression (Kumar *et al.* 2010; Awasthi *et al.* 2012). In the current research, *OsAPC6*, as a key gene involved in the development of female gametophytes, was significantly hypo-methylated in the promoter region of *fsv1* compared with Gui99. Moreover, RNA-Seq revealed that the *OsAPC6* gene was up-regulated in the meiosis stage and mature embryo sac stage and down-regulated in the mitosis stage in *fsv1* (Yang *et al.* 2016). Thus, we speculate that changes in the expression levels of the *OsAPC6* gene may affect the formation of female gametophytes. In *Lactuca sativa*, the concentration and distribution of calmodulin are critical for the occurrence of megaspore (Qiu *et al.* 2008). In our study, several key genes in the calmodulin signaling pathway, such as *OsCam3*, LOC_Os10g27170 (calmodulin-binding family protein), LOC_Os08g44670 (calmodulin-binding family protein), and LOC_Os09g13890 (plant calmodulin-binding protein-related), exhibited significant differences in methylation in *fsv1* ovules compared with Gui99. These differential methylation regions may affect female gametophyte fertility by regulating the expression of key genes in the calmodulin signaling pathway. Moreover, previous studies have shown that cytochrome P450 is a metabolic enzyme that is widely found in plants and plays a key role in regulating the growth and development of ovules (Adamski *et al.* 2009; Ma *et al.* 2015). In *Arabidopsis*, a CYP450 enzyme *CYP85A1* is required for the initiation of female gametogenesis (Pérez-España *et al.* 2011), and the *cyp78a8 cyp78a9* loss-of-function mutant shows abnormal ovule development, resulting in abortion of the majority of female gametophytes in the mutant (Sotelo-Silveira *et al.* 2013). In the present study, *CYP97A4* (LOC_Os02g57290), *CYP709C9* (LOC_Os07g23570), and several putative cytochrome P450 family genes (such as LOC_Os01g52800, LOC_Os02g09330, LOC_Os03g25150, LOC_Os06g43510, LOC_Os11g04310, among others) were differentially methylated in *fsv1* and Gui99 ovules, of which more DMGs were hypo-methylated in *fsv1*. Accordingly, the expression levels of *CYP97A4* and LOC_Os06g46680 were down-regulated, while LOC_Os06g43510, LOC_Os03g25150, LOC_Os07g29960, LOC_Os02g09400, and LOC_Os02g36280 were up-regulated in *fsv1* ovules (Yang *et al.* 2016), and these differentially expressed CYP genes may be associated with female gametophyte fertility.

SNF2 family proteins are ATP-dependent chromatin-remodeling factors that control many aspects of DNA events such as transcription, replication, homologous recombination, and repair. In *Arabidopsis*, many genes in the SNF2 family have been shown to be involved in ovule and embryo development (Bleuyard *et al.* 2004; Huanca-Mamani *et al.* 2005). Embryo sac development of the *atrad50* mutant is severely damaged, and this mutant does not produce any functional megaspores or viable gametes (Bleuyard *et al.* 2004). An SNF2 family gene, *CHR11*, encodes a chromatin-remodeling protein that is abundantly expressed during female gametogenesis in *Arabidopsis*, and specific degradation of the endogenous *CHR11* mRNA by RNA interference (RNAi) results in an increased frequency of ovule abortion and defective female gametophytes prior to completion of the mitotic haploid nuclear divisions (Huanca-Mamani *et al.* 2005; Yang *et al.* 2010). In rice, the SNF2 family of proteins exhibits a similar structure and function to *Arabidopsis thaliana* (Hu *et al.* 2013). In our study, *CHR721* and *CHR722* belonged to the SNF2 gene family, were hyper-methylated in intron of *fsv1* ovules compared with Gui99, and the expression levels of *CHR721* and *CHR722* were generally down-regulated (Yang *et al.* 2016). Thus, we speculate that these methylation differences in genes probably cause changes in expression levels and are likely to be closely related to female gametophyte abortion in *fsv1*.

In addition, TFs are significantly involved in plant female sexual reproduction as an important regulatory factor in gene expression (Bencivenga *et al.* 2012; Makkena *et al.* 2012). Studies have shown that DNA methylation can regulate the expression of TF genes in floral organ differentiation and development in plants (Sheldon *et al.* 1999; Zhao *et al.* 2005). Changes in the methylation status of some TF genes may affect female gametophyte fertility-related gene expression. In *Arabidopsis*, the TF SPL is required for cytokinin and auxin signaling during ovule development (Bencivenga *et al.* 2012). In rice, *OsSPL1* encodes long-chain base sphingosine-1-phosphate lyase, which acts as a signaling mediator in regulating programmed cell death (Zhang *et al.* 2014). In our study, two genes in the SPL TF family, *OsSPL1* and *SPL28*, showed significant differences in methylation between *fsv1* and Gui99 ovules. We hypothesize that changes in the methylation pattern of these two genes may cause programmed cell death during ovule development, leading to female gametophyte abortion. The AP2 (APETALA2) multigene family can be divided into AP2 and ANT groups, including developmentally and physiologically important TFs (Shigyo *et al.* 2006). In *Arabidopsis*, the *AP2* gene is expressed in egg and synergistic cells, and strong *ant* mutants have ovules that fail to form integuments or a female gametophyte (Klucher *et al.* 1996; Wang *et al.* 2009). In our study, three AP2 family genes, *OsHAP2A*, *OsHAP2D*, and *OsDREB1G*, were significantly hyper-methylated in promoter regions in *fsv1* compared with Gui99, and this high level of methylation in the promoter region may lead to a decrease in the gene expression level, which probably affects ovule development and female gametophyte fertility.

It is well known that plant hormones play essential parts in plant growth and development (Durbak *et al.* 2012). The available studies have shown that plant hormones are involved in ovule development (Bencivenga *et al.* 2012; Cheng *et al.* 2016). Auxin is one of the most important plant hormones and is involved in a number of biological processes of plant growth, development, and reproduction. Previous studies have shown that the expression of auxin biosynthesis and transport-related genes plays an important role in the formation of fertile female gametophytes (Panoli *et al.* 2015). ARF is a very important auxin response gene family, and the gene expression of this family can respond to changes in auxin (Ellis *et al.* 2005; Wang *et al.* 2007). In *fsv1* and Gui99 ovules, *OsARF2* was significantly hypo-methylated in *fsv1* compared with Gui99, and its expression level was up-regulated during meiosis (Yang *et al.* 2016). In contrast, *PIN* genes encode efflux carrier proteins of the phytohormone auxin in rice (Wang *et al.* 2009; Miyashita *et al.* 2010), while functional megaspore development is discontinued during mitosis to mutate the vast majority of female gametophytes in the *PIN1* mutant (*pDEFH9:amiPIN1*) of *A. thaliana* (Ceccato *et al.* 2013). We found that several *PIN* genes showed differential methylation in our analysis, among which *OsPIN8* was significantly hyper-methylated in *fsv1*, and their expression levels were up-regulated during meiosis and mitosis (Yang *et al.* 2016). The differential methylation of these two gene families, resulting in gene expression, may result in changes in the female gametophyte fertility of *fsv1*. The gibberellin class of plant hormones plays a role in many processes during plant development, including seed germination, cell elongation, flowering, and fruit setting (De Jong *et al.* 2009). Recent studies have shown that the gibberellin level is significantly reduced in abortive hazelnut compared with wild-type ovules (Cheng *et al.* 2016). In our study, two gibberellin oxidase genes, *OsGA2ox3* and *OsGA2ox4*, were significantly differentially methylated in exon regions in *fsv1* compared with Gui99 ovules, leading to further changes in expression levels and abnormal ovule development. In plants, many physiological and developmental processes, such as seed germination, flower senescence, and fruit ripening, are controlled by ethylene (Rieu *et al.* 2003; Linkies and Leubner-Metzger 2012). Ethylene also plays a significant role in ovule development and female gametophyte fertility (Tsai *et al.* 2008; Clark *et al.* 2010). We found that an ethylene-sensitive gene, *OsRTH2*, and an ethylene-forming enzyme gene, *OsACO3*, were significantly hypo-methylated in *fsv1*. The RNA-Seq data showed that the two genes were up-regulated and then down-regulated during ovule development (Yang *et al.* 2016), suggesting that these changes might be associated with female gametophyte abortion. Furthermore, jasmonic acid (JA) signaling has been well studied in *Arabidopsis*, and most reports have focused on the role of JA in biological pathways such as stress resistance, trichome initiation, and anthocyanin accumulation. However, there is some evidence that JA signaling pathways are involved in female sexual reproduction. For example, changes in the expression of key genes involved in jasmonate signaling pathways affect the formation of lint and fluff fibers in cotton ovules (Kim *et al.* 2015; Hu *et al.* 2016). In hazelnut, the JA signaling pathway is involved in ovule abortion (Cheng *et al.* 2016), and the key enzyme in the synthesis of JA precursors in the JA signaling pathway, AOC protein, is specifically expressed in ovules of tomato (Hause *et al.* 2000, 2003). In our study, several key genes in the rice jasmonate pathway, such as *OsJAZ3*, *OsJAZ14*, *OsJAZ15*, and *OsJAZ27*, were significantly differentially methylated and expressed in *fsv1* ovules, and we speculated that these changes likely affected the development of the female gametophyte.

The miRNAs play an essential role in the growth and development of plants, including sexual reproduction (Yang *et al.* 2013; Ding *et al.* 2016). Many studies have demonstrated that changes in the expression of key genes involved in important signaling pathways during ovule development can affect the fertility of female gametophytes (Olmedo-Monfil *et al.* 2010; Tucker *et al.* 2012). MiRNAs play an important regulatory role in gene expression, and their targeted genes are involved in multiple metabolic pathways during ovule development (Xie *et al.* 2015). In plants, the SBP-box genes are key genes regulating sexual reproductive growth (Shikata *et al.* 2009). In tomato, miR156 can regulate pistil development by targeting the SBP-box gene (Silva *et al.* 2014). In this study, the promoter region of the miR156i gene was hyper-methylated in *fsv1* ovules, which may inhibit the expression of miR156i and thus affect the expression of the SBP-box genes related to the formation of fertile female gametophytes. As mentioned above, auxin plays an important role in the regulation of plant ovule development. When some auxin-related genes are abnormally expressed in ovules, the female gametophyte becomes stagnant,and the cells differentiate abnormally, seriously affecting the female gametophytes (Ceccato *et al.* 2013; Panoli *et al.* 2015). In *Arabidopsis*, miRNAs can regulate the fertility of female gametophytes by regulating changes in auxin-related gene expression, in which miR160 targets multiple ARF family genes. Abnormal expression of miR160 alters the expression of auxin-related genes, leading to smaller petals and early maturation of *A. thaliana*, among other features (Mallory *et al.* 2005). In addition, miR167 controls the development of ovules and anthers by targeting the TF genes *ARF6* and *ARF8*, and its abnormal expression in pistils and stamens causes abortion (Wu *et al.* 2006). In the present study, the promoter regions of the miR160c-3p, miR160c-5p, miR167b, and miR167f genes were significantly hypo-methylated, potentially leading to the abnormal expression of miR160 and miR167 and thus affect the synthesis of auxin, which further influences female gametophyte formation. *PHB*, *PHA*, and *CNA*, which are members of the HD-ZIP TF family, are key genes in the regulation of ovule development. In the *A. thaliana* mutant *phb-13phv-11cna-2*, ovule development exhibits severe defects that lead to the abortion of the majority of female gametophytes (Prigge *et al.* 2005). Experiments have shown that miR166 targets the expression of HD-ZIP family genes in plants (Zhou *et al.* 2015). In our study, the promoter methylation statuses of the miR166b-3p, miR166b-5p, miR166c-3p, miR166d-3p, miR166d-5p, miR166e-3p, and miR166e-5p genes were abnormal in *fsv1* ovules, potentially leading to changes in the expression of target genes and ultimately affecting the development of ovules and causing female gametophyte abortion. In rice, miR172 targets and regulates AP2 family member genes, and the floral organs display serious developmental defects that lead to a significant reduction in fertility in miR172-overexpressing mutant plants (Zhu *et al.* 2009). In our study, the miR172a gene promoter region was hyper-methylated in *fsv1* compared with Gui99, potentially affecting the expression levels of AP2 family genes related to female gametophyte fertility. Rice miRNAs are closely related to fertility, and DNA methylation is very important for the regulation of miRNAs, supporting the importance of further studies.

In summary, we have systematically investigated changes in DNA methylation levels in the highly female-sterile and wild-type rice ovules by high-throughput sequencing, and we have obtained a large number of DMRs and genes. Based on the whole-genome DNA methylation map, hypo-methylation was observed in *fsv1*, and the DMGs were involved in many pathways, such as plant hormone metabolism, DNA replication and transcriptional regulation, and glycerophospholipid metabolism. We propose that female gametophyte fertility is a complex process involving multiple pathways, and that changes in methylation may affect this process. We also found that some auxin-related genes, TF genes, and miRNA gene promoter regions displayed significant differences in methylation in *fsv1* and Gui99 ovules, and these DMGs are being further examined in our laboratory. In conclusion, the above studies reveal the genome methylation pattern of ovules of the female-sterile rice line *fsv1* and fertile rice line Gui99, providing important clues for further analyses of the molecular mechanism of female gametophyte fertility.

## Supplementary Material

Supplemental material is available online at www.g3journal.org/lookup/suppl/doi:10.1534/g3.117.300243/-/DC1.
