# Supplementary material for: Genome-Wide Analysis of DNA Methylation During Ovule Development of Female-Sterile Rice fsv1
Source: G3 (Bethesda). 2017 Sep 6;7(11):3621–35. doi: 10.1534/g3.117.300243 (PMC5677159; doi:10.1534/g3.117.300243)
Supplement: Supplementary file 1 [file 3621FigureS1.pdf]

Gui99

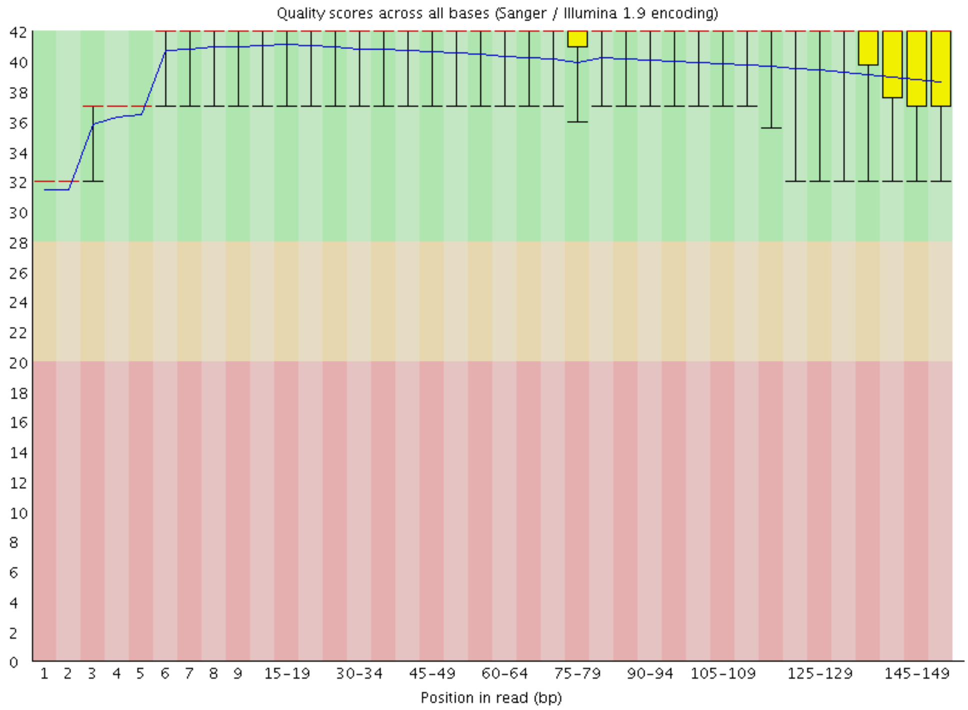

fsv1

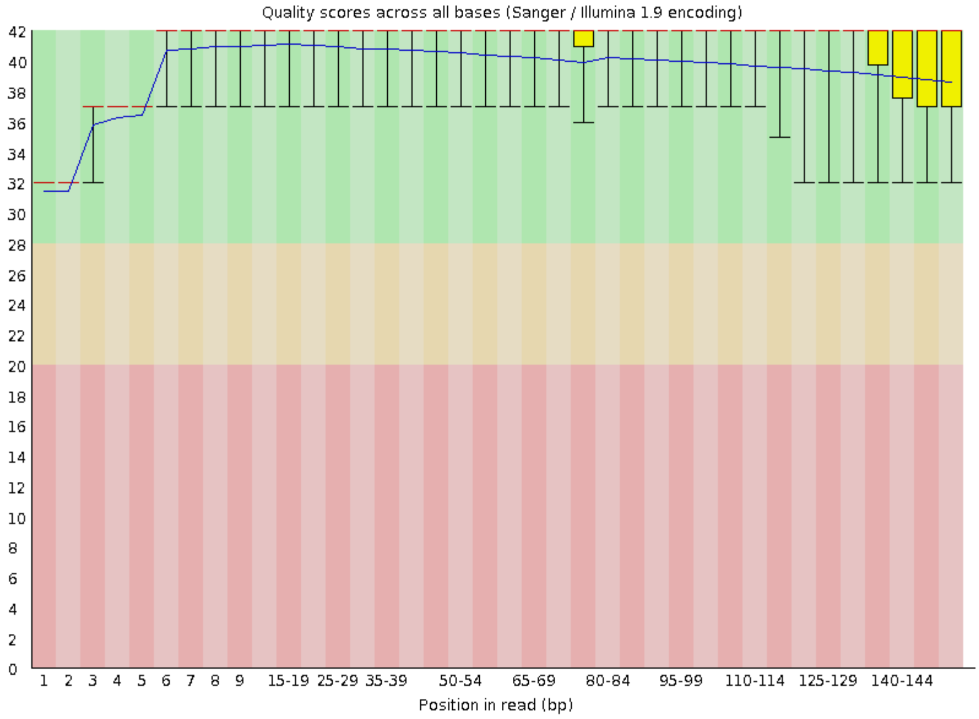

Figure S1 Use FastQC to assess the quality of the WGBS raw data in *fsv1* and Gui99 ovules. Q30 > 80% indicate high quality sequencing.
