# Supplementary material for: Genome-Wide Analysis of DNA Methylation During Ovule Development of Female-Sterile Rice fsv1
Source: G3 (Bethesda). 2017 Sep 6;7(11):3621–35. doi: 10.1534/g3.117.300243 (PMC5677159; doi:10.1534/g3.117.300243)
Supplement: Supplementary file 2 [file 3621FigureS2.pdf]

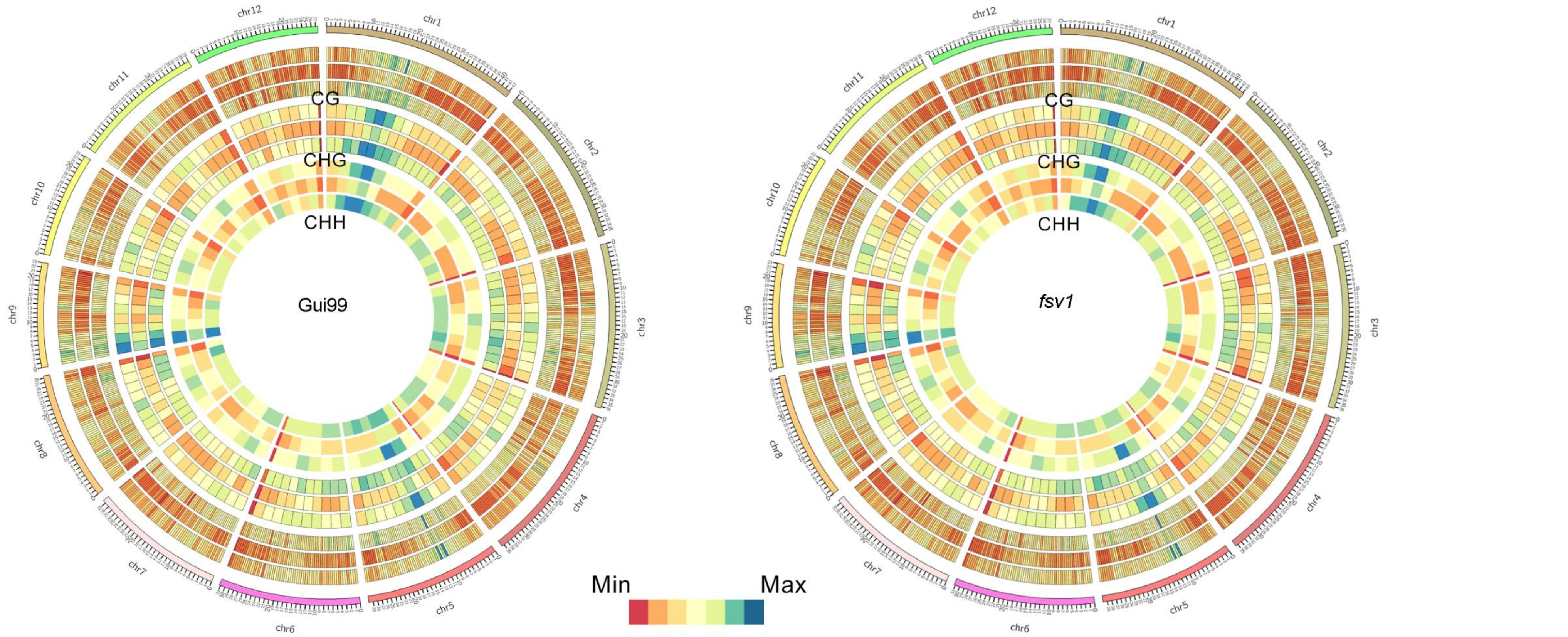


**Figure S2** Circos diagram showing the methylation level of CG, CHG and CHH in genome-wide of two rice lines ovules. In this figure, the entire rice chromosomes are displayed by the first outer ring. Nine concentric rings are divided into three parts, every three rings from outside to inside represent CG, CHG, CHH sites in each chromosome. Colors represent (from red to blue) the methylation levels of all detected genome elements.
