# Supplementary material for: Genome-Wide Analysis of DNA Methylation During Ovule Development of Female-Sterile Rice fsv1
Source: G3 (Bethesda). 2017 Sep 6;7(11):3621–35. doi: 10.1534/g3.117.300243 (PMC5677159; doi:10.1534/g3.117.300243)
Supplement: Supplementary file 3 [file 3621FigureS3.pdf]

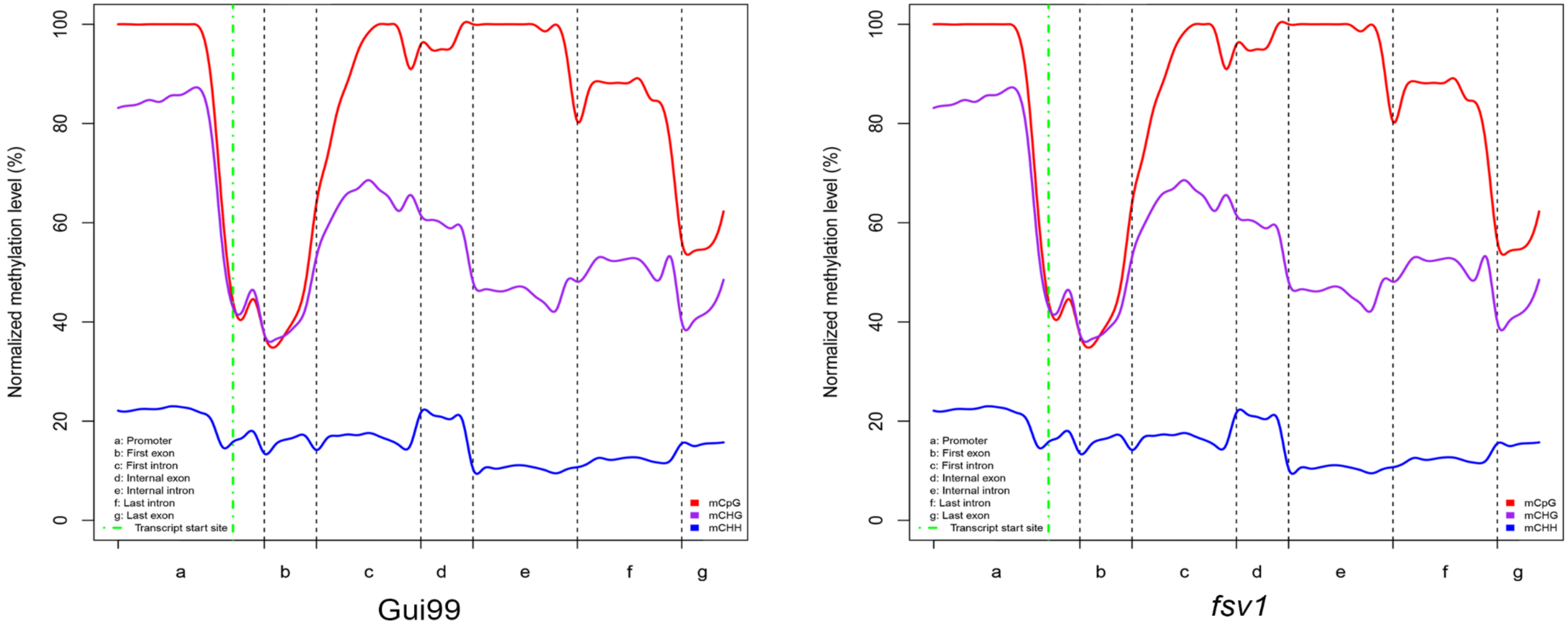

Figure S3 The standard methylation status of the gene elements in *fsv1* and Gui99. In the figure, gene was divided into seven elements: promoter, first exon, first intron, internal intron, internal exon, last intron, last exon. According to the methylation status of all the genes in each sample, normalized methylation level of CG, CHG, CHH sites was calculated in each gene elements.
