# Supplementary material for: Genome-Wide Analysis of DNA Methylation During Ovule Development of Female-Sterile Rice fsv1
Source: G3 (Bethesda). 2017 Sep 6;7(11):3621–35. doi: 10.1534/g3.117.300243 (PMC5677159; doi:10.1534/g3.117.300243)
Supplement: Supplementary file 4 [file 3621FigureS4.pdf]

(A)

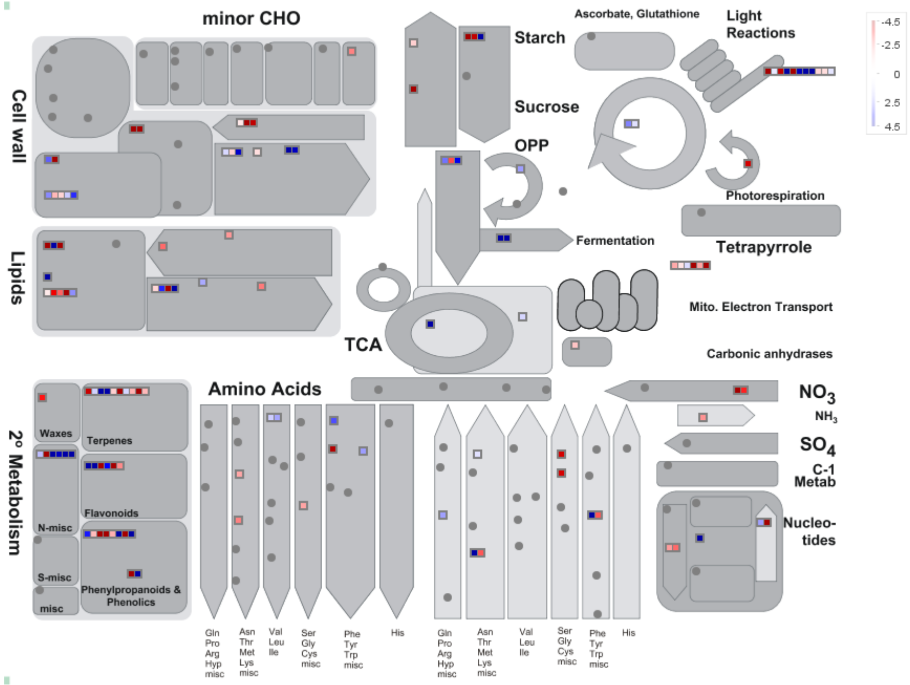

(B)

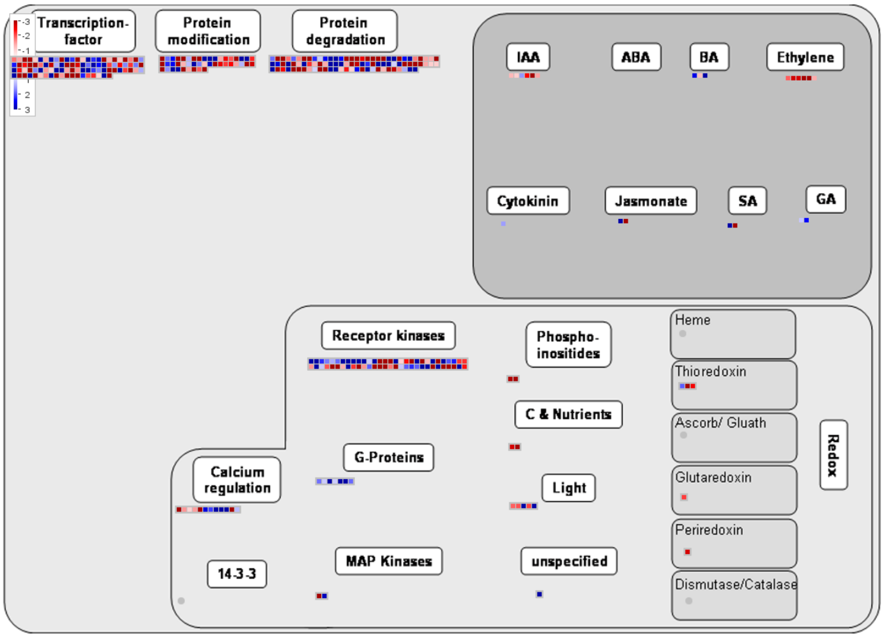

Figure S4 MapMan overview of DMGs enriched in (A) cellular metabolism and (B) regulation of *fsv1* and Gui99. The color key indicated the value of $\log_2$ fold change. Red meant hypomethylated genes and blue meant hypermethylated genes.
