# Supplementary material for: Genome-Wide Analysis of DNA Methylation During Ovule Development of Female-Sterile Rice fsv1
Source: G3 (Bethesda). 2017 Sep 6;7(11):3621–35. doi: 10.1534/g3.117.300243 (PMC5677159; doi:10.1534/g3.117.300243)
Supplement: Supplementary file 5 [file 3621FigureS5.pdf]

LOC_Os08g30660 : NB-ARC domain-containing disease resistance protein.

Gui99

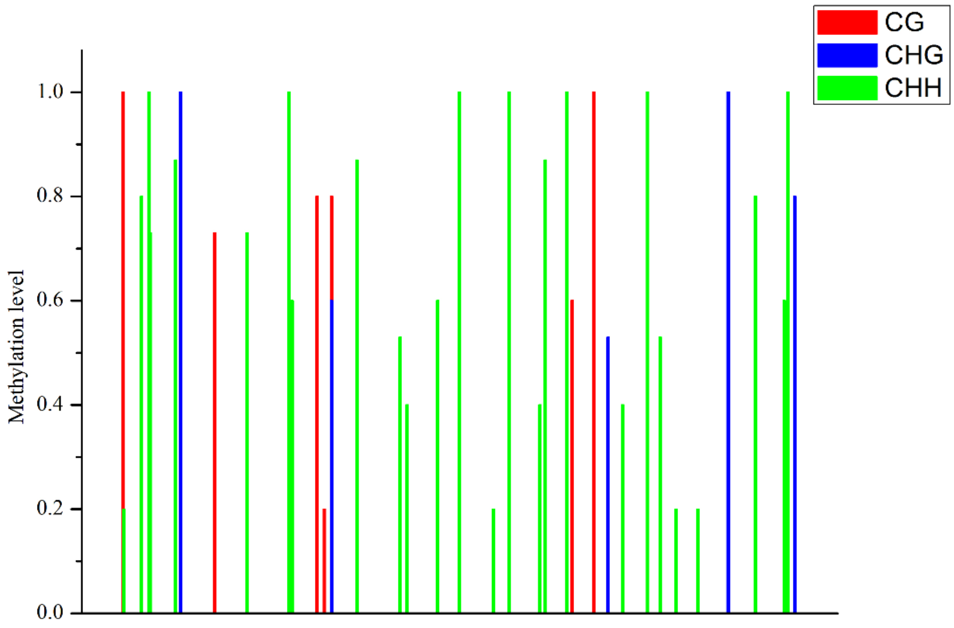

fsv1

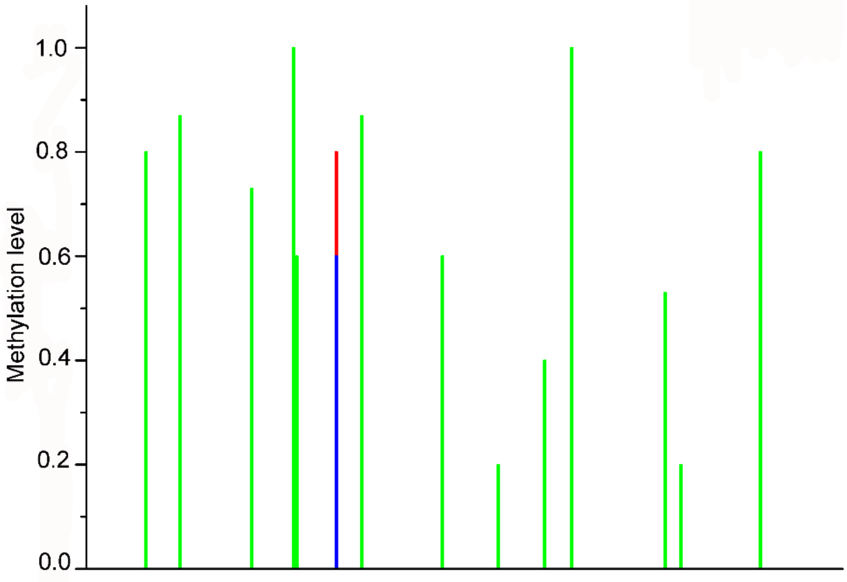

LOC_Os12g41890 : amino acid transporter 1.

Gui99

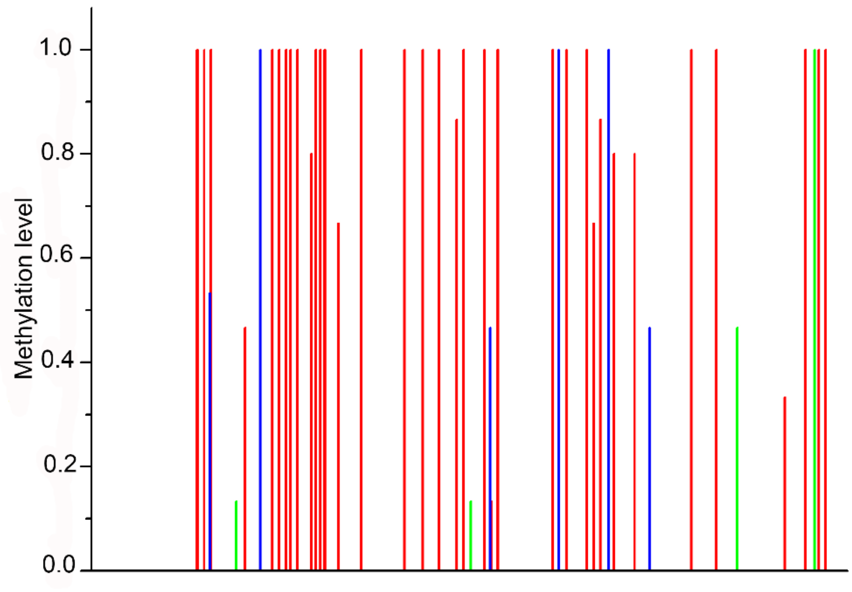

fsv1

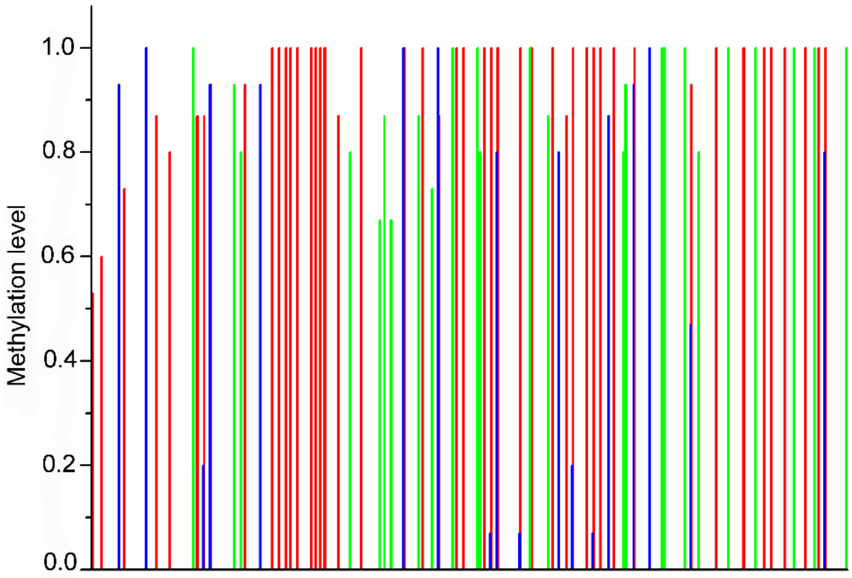

LOC_Os06g47350 : RNA polymerase I specific transcription initiation factor RRN3 protein.

Gui99

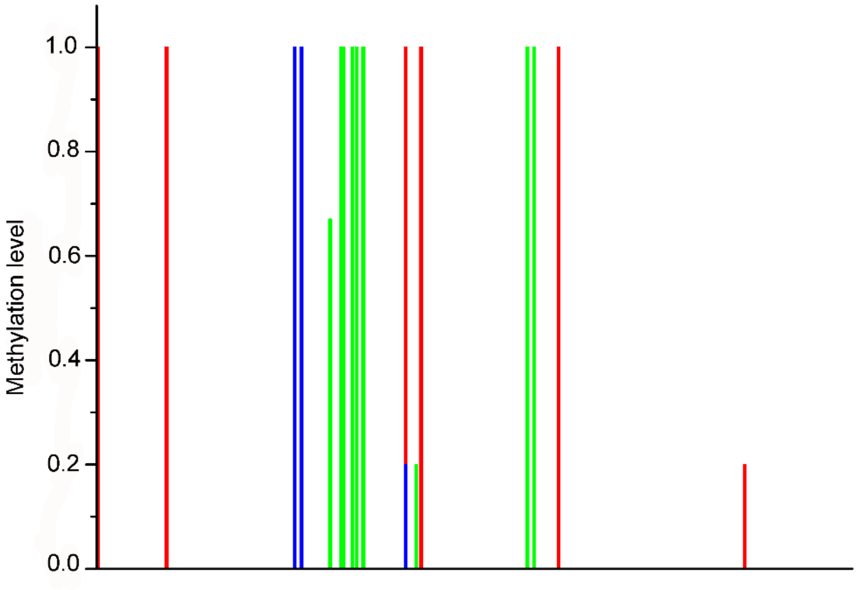

fsv1

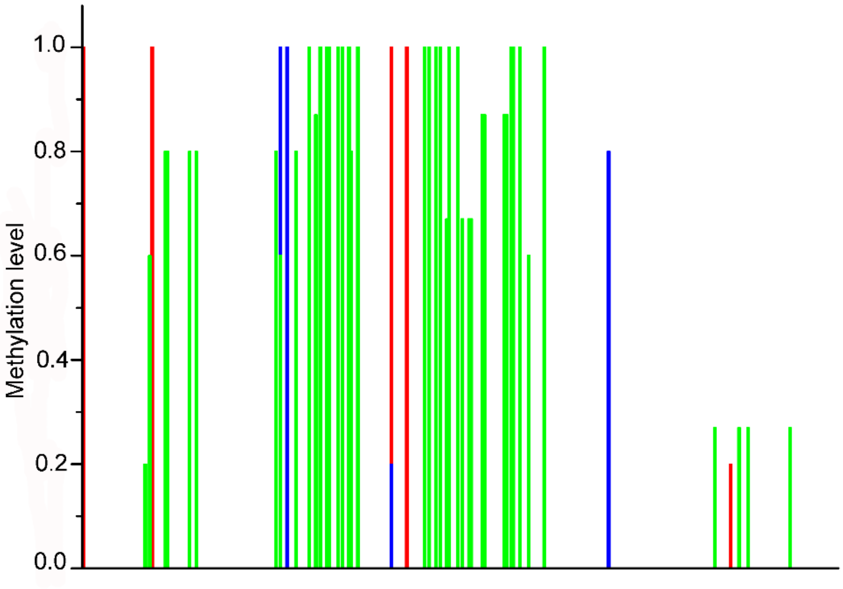

LOC_Os09g38080 : NA.

Gui99

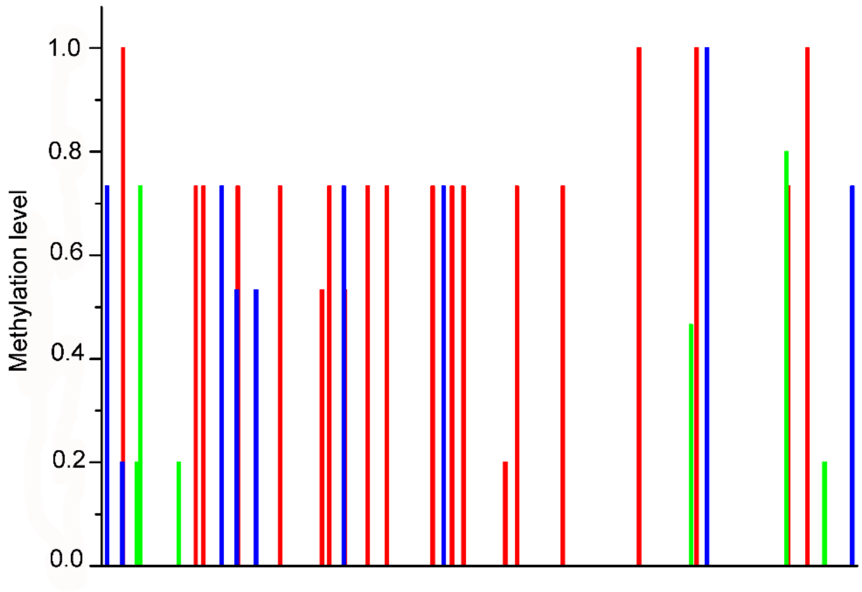

fsv1

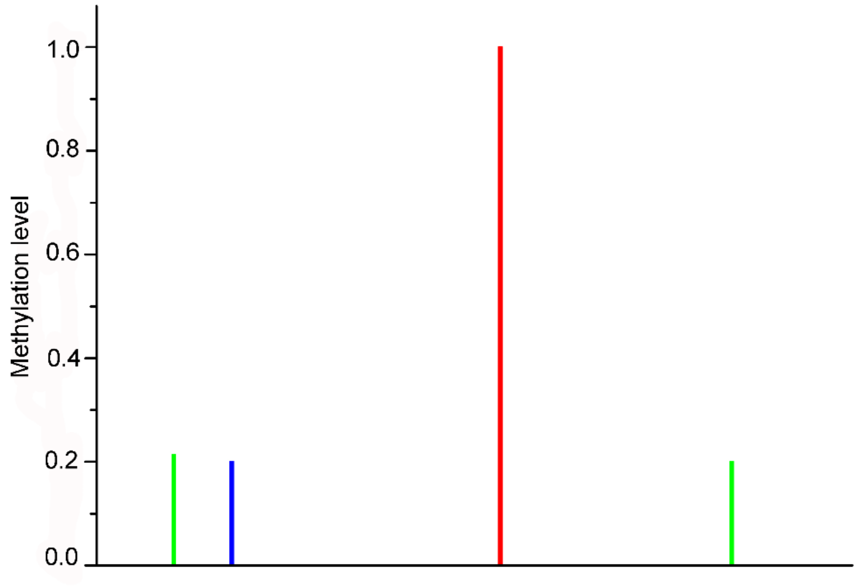

LOC_Os01g17130 : LRR and NB-ARC domains-containing disease resistance protein.

Gui99

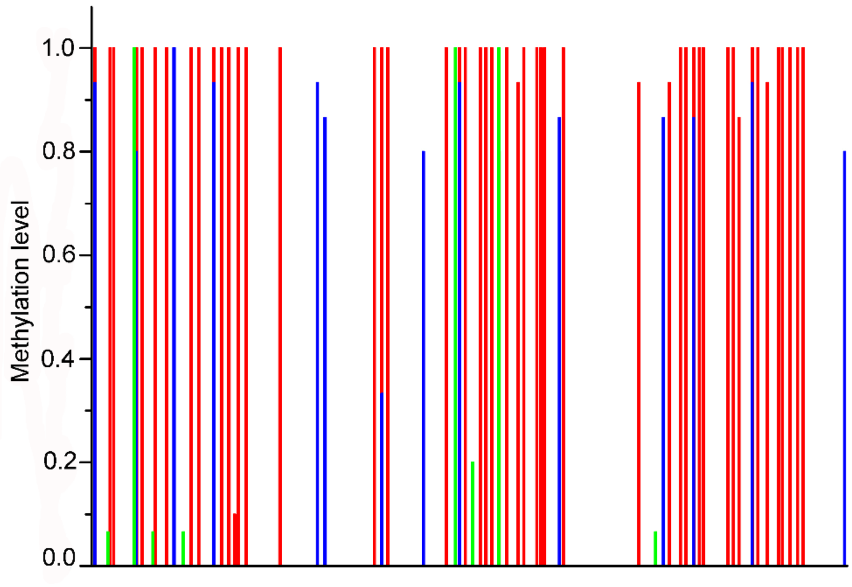

fsv1

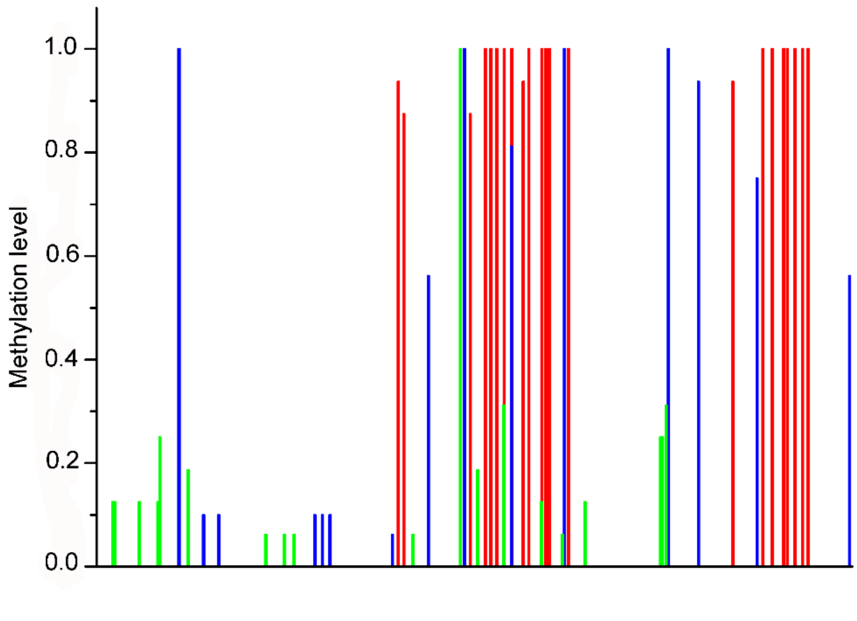

LOC_Os10g34490 : Nucleotide-sugar transporter family protein.

Gui99

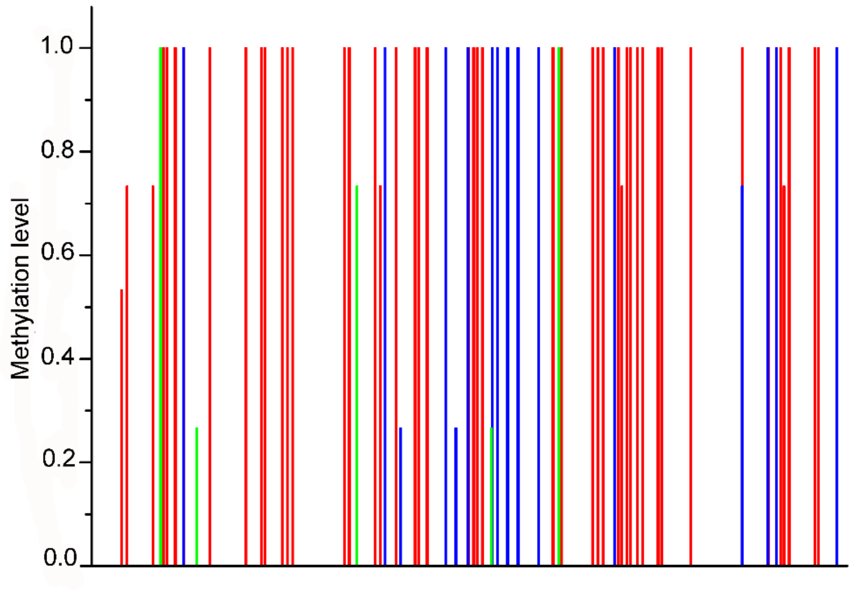

fsv1

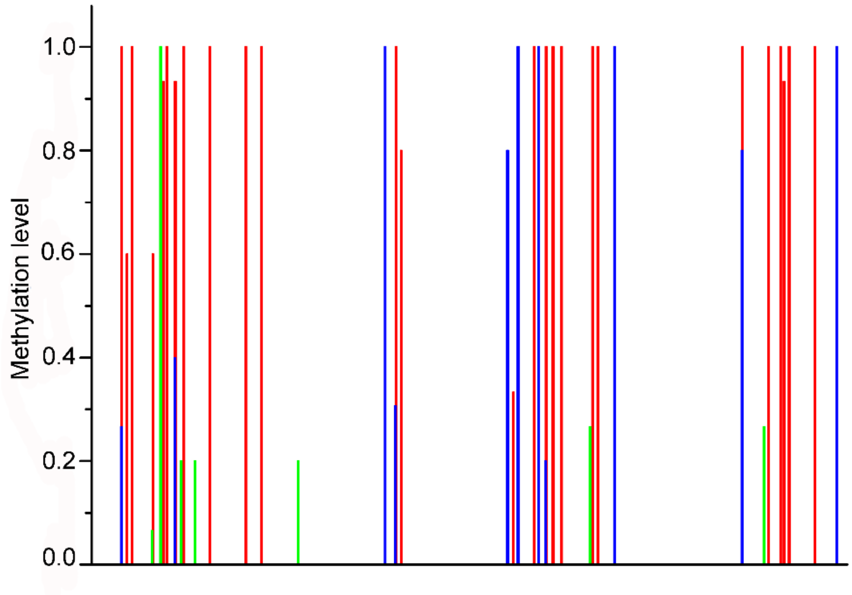

LOC_Os12g08300 : glycosyltransferase family protein 2.

Gui99

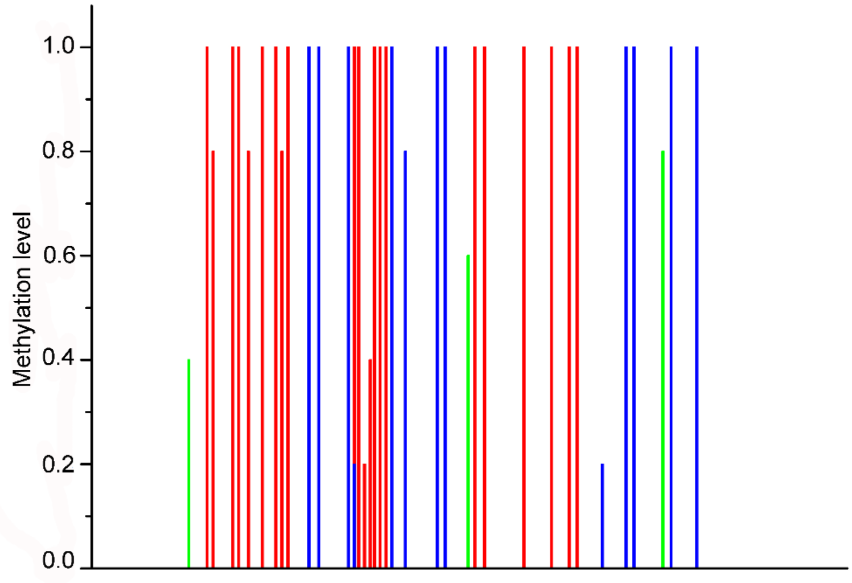

fsv1

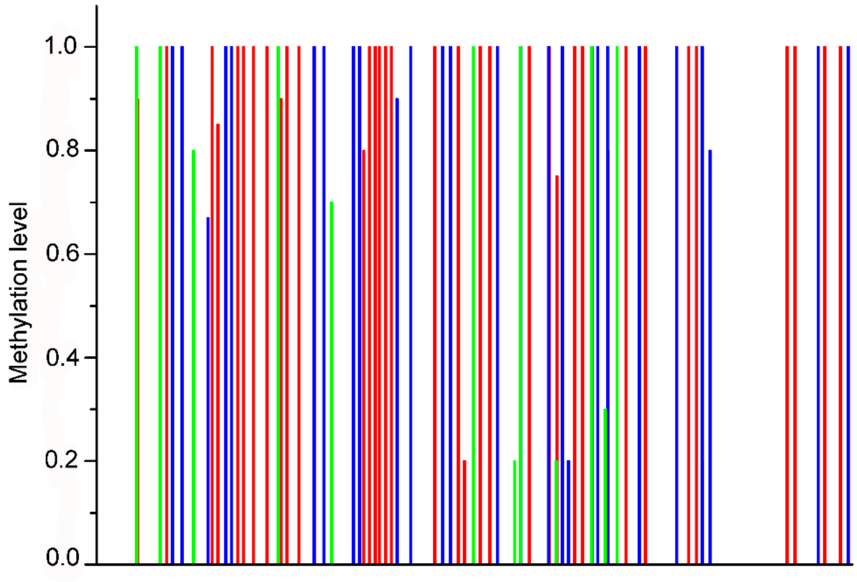

LOC_Os11g32260 : Glycosyl hydrolase family 38 protein.

Gui99

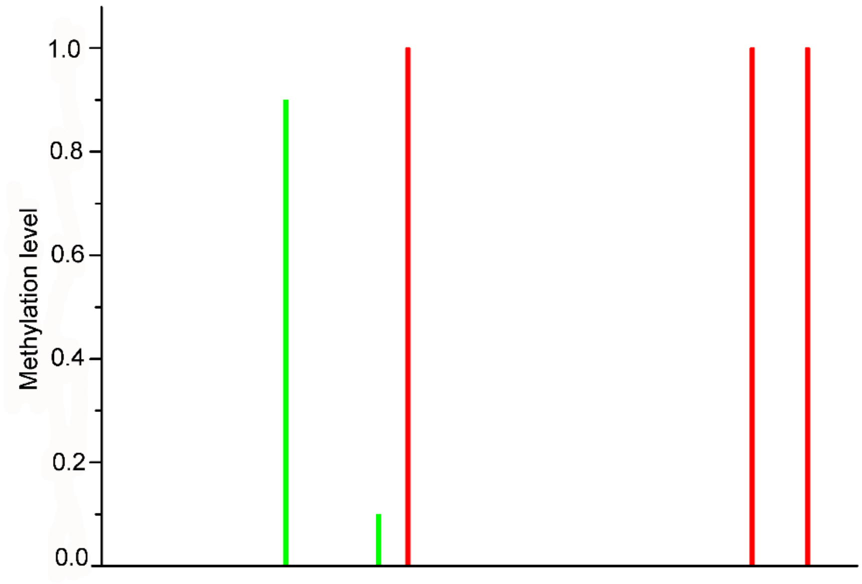

fsv1

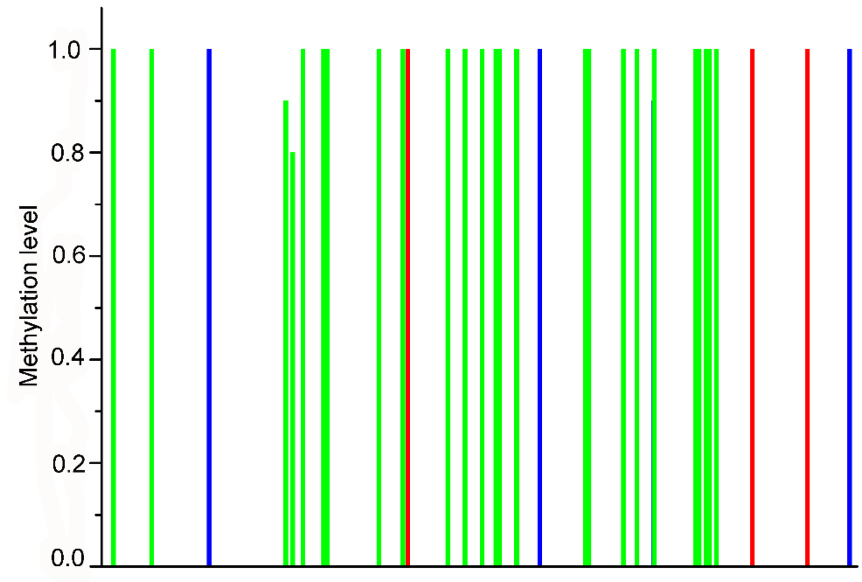

LOC_Os12g31180 : retrotransposon protein.

Gui99

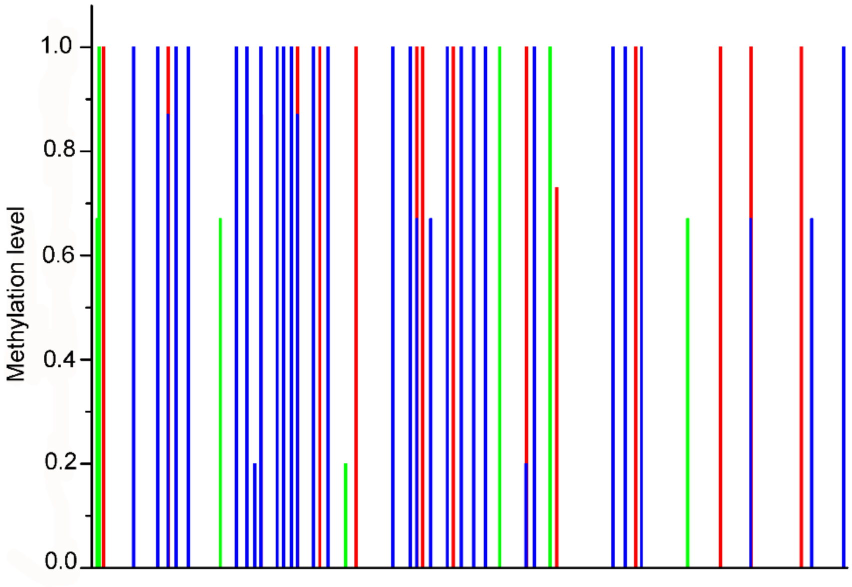

fsv1

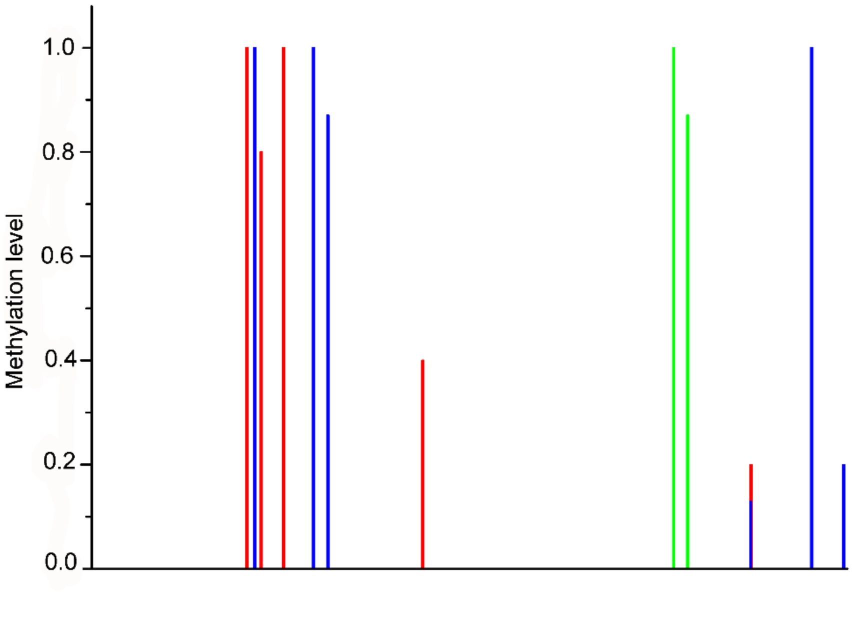

LOC_Os05g16680 : retrotransposon protein.

Gui99

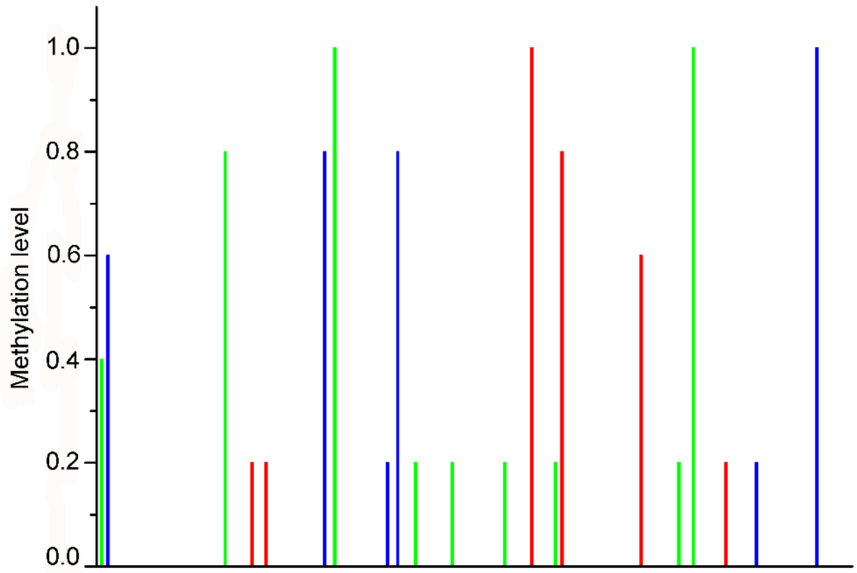

fsv1

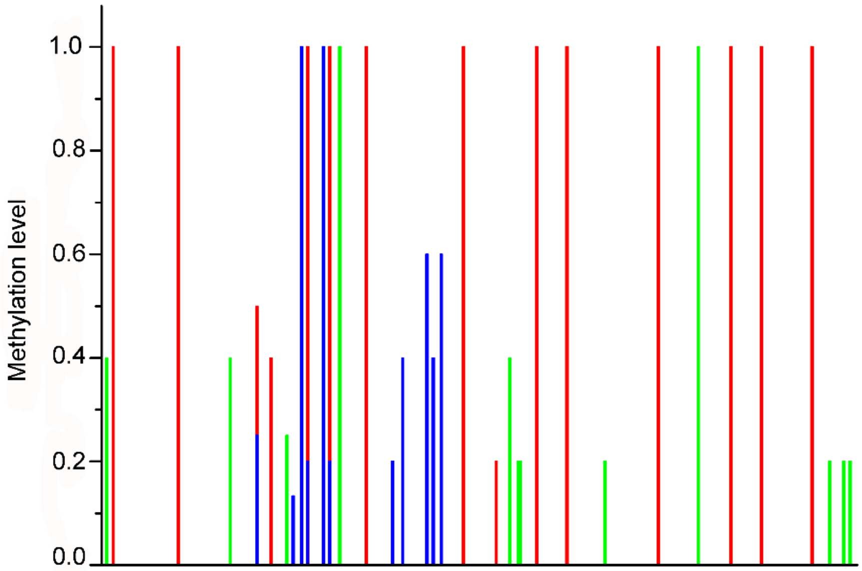

Figure S5 Bisulfite sequencing for ten selected DMR-associated genes.
